# DNA Barcoding and Phylogenomic Analysis of the Genus *Fritillaria* in China Based on Complete Chloroplast Genomes

**DOI:** 10.3389/fpls.2022.764255

**Published:** 2022-02-25

**Authors:** Qi Chen, Haisu Hu, Dequan Zhang

**Affiliations:** ^1^College of Pharmacy, Dali University, Dali, China; ^2^Institute of Materia Medica, Dali University, Dali, China

**Keywords:** *Fritillaria*, chloroplast genomes, DNA barcoding, species discrimination, phylogenomic analysis, molecular dating

## Abstract

The *Fritillaria* is an extremely complicated genus in taxonomy and phylogeny, which contains numerous medicinal species in China. Both traditional characteristic-based taxonomy and universal DNA barcodes (ITS, *trnH*-*psbA*, and *rbcL*) are difficult to effectively identify the species. Here, we generated a large dataset of chloroplast genomes from multiple accessions per species of *Fritillaria* to evaluate their effectiveness in species discrimination. Moreover, phylogeny of species in China was explored based on the complete chloroplast genomes, and then divergence times of each node were estimated. The results showed that all 21 species in *Fritillaria* here (including two suspicious species) could be correctly discriminated using cpDNA genomes except *F. cirrhosa*, which suggested that DNA super-barcode could greatly enhance species discriminatory resolution for complicated genera. Furthermore, four regions (*ycf1*, *matK*-*trnG-GCC*, *rpoC1*, and *matK*) gained remarkably higher resolution than that of other plastid regions, but only *matK* might be suitable to identify *Fritillaria* species in consideration of its lengths. Phylogenomic analysis showed that the subgenus *Fritillaria* in China was divided into four major clades with obvious geographic structure. Among them, Clade I, mainly distributed in southwest China, was a young and complicated group. Moreover, according to the analysis, taxonomic treatments of the two suspicious species, namely “*F. omeiensis*” and “*F. hupehensis*” in *Flora of China* (2000) are questionable and might need further revision. Molecular dating revealed that both origin and divergence of subgenus *Fritillaria*, as well as its four major clades, were significantly associated with geological and climatic fluctuations during the Middle to Late Miocene. This study would enrich case studies of DNA super-barcode and provide new insights on speciation, lineage diversification, and biogeography of the *Fritillaria* in China.

## Introduction

Accurate identification of a species is an essential condition for the sustainable utilization of biological resources. It could help relevant management departments to formulate corresponding protection and management measures for a certain group (Parveen et al., 2012; Sembiring et al., 2015; Liu et al., 2018). Traditional taxonomy is generally performed based on morphological characteristics, which depend upon taxonomists possessing rich knowledge on taxonomy and conducting careful analysis of specimens (Godfray, 2002; Li et al., 2011). However, in some cases, when the amounts of specimens (e.g., specimens of bees or fish) are very abundant in a study (Hou et al., 2018; Gueuning et al., 2019), the analysis of morphology tends to be lengthy and expensive, which could result in serious decreasing of the reliability of species identification. Moreover, if available specimens belong to a complicated genus or they are sub-optimal (e.g., sterile, juvenile, and/or poor), accurate identification in a way also might be ineffective or even impossible for the traditional method (Gonzalez et al., 2009; Yan et al., 2015; Liu et al., 2017, 2018).

As a supplementary tool for morphological taxonomy, DNA barcoding, using short and standardized DNA fragments, was proposed by Paul Hebert in 2003, and quickly became an efficient method for species identification and discovery (Hebert et al., 2003; Barrett and Hebert, 2005; Chase et al., 2005; Li et al., 2011; Schoch et al., 2012). The mitochondrial gene cytochrome oxidase 1 (COI) has been proven to be effective and reliable for species identification on animals as the unique core DNA barcode (Hebert et al., 2003; Barrett and Hebert, 2005; Hajibabaei et al., 2006; Costa et al., 2007; Kim et al., 2012). In land plants, there is no single barcode that could successfully identify most species; therefore, multiple regions, including three plastid regions (e.g., *rbcL*, *matK*, and *trnH*-*psbA*) and nrDNA ITS are widely regarded as universal DNA barcodes (Kress et al., 2005; CBOL Plant Working Group, 2009; Hollingsworth et al., 2010; Hollingsworth, 2011; Li et al., 2011; Coissac et al., 2016). However, for complicated groups in taxonomy, the barcodes might be futile, especially in recently diverged and rapidly radiated taxa (Hollingsworth, 2011; Li et al., 2011; Zhang et al., 2011; Liu et al., 2016). Therefore, there is an urgent need to develop new and robust approaches that could satisfy requirements of identifying the complicated ones. Luckily, rapid development of the next-generation DNA sequencing (NGS) technology provides the possibility for species discrimination at the genome level (Fu et al., 2019; Ji et al., 2019).

The chloroplast genome, namely plastome, is a perfect source for resolving the tree of life and delimiting species entity in angiosperms (Gitzendanner et al., 2017; Fu et al., 2019). Numerous studies showed that the chloroplast genome was a robust and appropriate tool that could provide much better ability than the universal regions on revealing phylogeny and evolutionary history of plants (Li et al., 2019; Nie et al., 2019). DNA super-barcode, using complete chloroplast genome sequence, exhibits a powerful ability to identify the closely related species, due to their abundant genetic variation in contrast with the four universal barcodes (Wu et al., 2010; Carbonell-caballero et al., 2015; Li et al., 2015; Chen et al., 2019; Fu et al., 2019; Jiao et al., 2019). Of course, there is no denying that DNA super-barcode is still faced with controversy and challenges in species identification, such as high sequencing cost, establishment of a rich cp-genome database, and difficulty in tracking species boundaries (Li et al., 2015). In recent years, along with quickly decreasing of the cost for the NGS sequencing and developing analysis methods for genomes, more and more cpDNA genomes are available in GenBank, thus a series of genera already have corresponding reference genomes for delivering species identification or phylogenomics (Li et al., 2015; Coissac et al., 2016; Gitzendanner et al., 2017; Fu et al., 2019; Ji et al., 2019). Similarly, some regions from the plastomes, selected as special barcodes are tested and used in the complicated taxa, such as *accD* and *rrn16*-*rrn23* for yew species (Fu et al., 2019) and *psbE*-*psbL* and *ndhA* intron for *Fagopyrum* (Huang et al., 2019). Nevertheless, for most of the genera, it is still insufficient to construct a reliable database due to limited reference genomes or specific fragments.

*Fritillaria* L. (Liliaceae) is a popular genus close to *Lilium* L., and it mainly grows in the temperate regions of the northern hemisphere. The genus was first established by Carolus Linnaeus in 1753 and then systematically revised by Baker in 1874, as well as Bentham and Hooker in 1883, Turrill and Sealy in 1980, Yibo Luo in 1993, and Rix in 2001 (Rønsted et al., 2005). According to Rix’s classification system, *Fritillaria* could be divided into eight subgenera, which includes approximately 140 species of perennial herbaceous plants all over the world (Rønsted et al., 2005; Day, 2018). In China, there are 24 species that were divided into three subgenera (*Fritillaria*, *Davidii*, and *Liliorhiza*), and about 10 species and 2 varieties are listed as original species of five medicines in Chinese Pharmacopoeia Commission [CPC] (2020), namely the so-called “Beimu,” which mainly relieves cough and eliminates phlegm, such as *F. cirrhosa* D. Don, *F. ussuriensis* Maxim., *F. walujewii* Regel, *F. thunbergii* Miq, and so on (Chen and Helen, 2000; Xiao et al., 2007). However, some species in *Fritillaria* are extremely complicated in taxonomy, especially the so-called “*Fritillaria cirrhosa* D. Don complex,” namely *F. cirrhosa* and its related species in southwest China (Luo and Chen, 1996a,b). *F. cirrhosa* is a complicated species in morphology which possesses diverse floral characteristics (e.g., color and plaques of tepals, lengths of stigma lobes, number of bracts, etc.); meanwhile, transitional variation among the related species further results in difficulty on delimiting the ones. A series of studies revealed that universal barcodes (*matK*, *rbcL*, *trnH*-*psbA*, ITS) or plastid region (*rpl16*) could not afford efficient identification for such a complicated genus (Zhang et al., 2016; Huang et al., 2018; Chen et al., 2020). Until now, complete chloroplast genomes have been adopted to explore phylogeny of *Fritillaria* species, and showed unambiguous relationships with highly supportive values, especially for the related species (Park et al., 2017; Bi et al., 2018; Li et al., 2018; Chen et al., 2019; Wu et al., 2021; Zhang et al., 2021). But these studies generally only cover minority species with few samples, so they could not represent the species well. Moreover, recent research illuminated *Fritillaria* was evolved in the Early Miocene (17∼26 Mya) and the subgenera *Fritillaria* of China was divided into three subclades, whereas the divergence times of these subclades are still ambiguous (Huang et al., 2018). Hence, it is necessary to adopt more cpDNA genomes to better represent the species, to construct a more reliable and comprehensive database. Then, we could further evaluate the ability of species discrimination of DNA super-barcode for *Fritillaria* species and reconstruct a more reliable phylogenetic tree with better representativeness.

Here, a large dataset of complete chloroplast genomes, newly obtained from multiple individuals per species for the *Fritillaria* in China, was adopted to construct a comprehensive database for analysis on species discrimination and phylogeny. This study aims to address the following questions: (i) As DNA super-barcode, is a complete chloroplast genome suitable to discriminate species in *Fritillaria*? Or, are there any specific plastid regions that could provide better choices? (ii) Could cpDNA genomes effectively reveal complicated phylogeny within the genus, especially *F. cirrhosa* and its closely related species? (iii) If there is a clear phylogenetic relationship among these species, what are the main time nodes of the important lineages of *Fritillaria* in China? The present study would provide further insight on super-barcode and broaden the horizon for phylogeny, as well as evolution of the important genus.

## Materials and Methods

### Material Sampling

A total of 73 individuals, collected from 21 species (including two suspicious species, namely “*Fritillaria omeiensis*” and “*F. hupehensis*”) in *Fritillaria* almost representing most of the species in China, were mainly sampled from their wild habitats. Some species, such as *F. pallidiflora*, “*F. hupehensis*,” and *F. thunbergii*, were obtained from cultivation bases of their main distributions. Fresh leaves, used as molecular materials, were sampled from healthy and mature individuals in fieldwork, and then dried by allochroic silicagel. For each species, 2∼6 individuals from one or two populations were sampled to represent genetic variation within species. Meanwhile, 3∼5 individuals in flowering or fruiting stages were dug and preserved as vouchers; meanwhile, geographic information of sampling locations was measured by Global Position System (GPS, Garmin). All voucher specimens were identified by Professor Dequan Zhang on the basis of morphological evidence according to *Flora of China* (Chen and Helen, 2000; Table 1 and Supplementary Figure 1). Then, these specimens of *Fritillaria* species were deposited at the Herbarium of Medicinal Plants and Crude Drugs of the College of Pharmacy, Dali University. Besides the newly obtained chloroplast genomes, 9 cpDNA genomes in NCBI databases (Supplementary Table 1), from four species in *Fritillaria*, were also downloaded and used for resolving phylogeny and estimation on divergence times in this study.

**TABLE 1:** Collecting information of the 21 species (including the suspicious species) in *Fritillaria* in China.

Species	Code	Locality	Latitude/Longitude	Altitude (m)	Voucher specimen	Accession number of plastome
*F. cirrhosa*	BM1-1	Lijiang, Yunnan, China	N27°03.570′/E100°14.130′	3,142	ZDQ15019	MH593342
*F. cirrhosa*	BM1-2	Lijiang, Yunnan, China	N27°03.570′/E100°14.130′	3,142	ZDQ15019	MH593343
*F. cirrhosa*	BM2-1	Shangri-La, Yunnan, China	N28°08.100′/E99°52.880′	4,212	ZDQ13053	MH244906
*F. cirrhosa*	BM2-2	Shangri-La, Yunnan, China	N28°08.100′/E99°52.880′	4,212	ZDQ13053	MH593344
*F. cirrhosa*	BM3-1	Basu, Xizang, China	N29°38.636′/E96°42.856′	4,480	ZDQ14027	MH593345
*F. cirrhosa*	BM3-2	Basu, Xizang, China	N29°38.636′/E96°42.856′	4,480	ZDQ14027	MH593346
*F. sichuanica*	BM5-1	Kangding, Sichuan, China	N30°03.162′/E101°43.434′	3,722	ZDQ13010	MN810967
*F. sichuanica*	BM5-2	Kangding, Sichuan, China	N30°03.162′/E101°43.434′	3,722	ZDQ13010	MN810968
*F. przewalskii*	BM6-1	Ganzi, Sichuan, China	N31°33.164′/E100°00.926′	3,682	ZDQ13018	MH244908
*F. przewalskii*	BM6-2	Ganzi, Sichuan, China	N31°33.164′/E100°00.926′	3,682	ZDQ13018	MH593347
*F. przewalskii*	BM7-1	Ganzi, Sichuan, China	N31°45.895′/E100°45.653′	4,047	ZDQ13029	MH593348
*F. przewalskii*	BM7-2	Ganzi, Sichuan, China	N31°45.895′/E100°45.653′	4,047	ZDQ13029	MH593349
*F. unibracteata*	BM8-1	Hongyuan, Sichuan, China	N32°10.532′/E102°30.686′	3,621	ZDQ13030	MH244909
*F. unibracteata*	BM8-2	Hongyuan, Sichuan, China	N32°10.532′/E102°30.686′	3,621	ZDQ13030	MH593350
*F. unibracteata*	BM9-1	Songpan, Sichuan, China	N32°53.419′/E103°30.390′	3,199	ZDQ13032	MH593351
*F. unibracteata*	BM9-2	Songpan, Sichuan, China	N32°53.419′/E103°30.390′	3,199	ZDQ13032	MH593352
*F. delavayi*	BM10-1	Lijiang, Yunnan, China	N27°03.520′/E100°11.810′	4,071	SS12-04	MH593353
*F. delavayi*	BM10-2	Lijiang, Yunnan, China	N27°03.520′/E100°11.810′	4,071	SS12-05	MH593354
*F. delavayi*	BM10-3	Lijiang, Yunnan, China	N27°03.520′/E100°11.810′	4,071	SS12-10	MH593355
*F. taipaiensis*	BM11-1	Wuxi, Chongqing, China	N31°33.860′/E109°06.490′	2,230	HCB1	MH244910
*F. taipaiensis*	BM11-2	Wuxi, Chongqing, China	N31°33.860′/E109°06.490′	2,230	HCB17	MH593356
*F. taipaiensis*	BM12-1	Foping, Shaanxi, China	N33°36.7′/E107°48.418′	1,470	ZDQ15017	MH593357
*F. taipaiensis*	BM12-2	Foping, Shaanxi, China	N33°36.7′/E107°48.418′	1,470	ZDQ15017	MH593358
*F. taipaiensis*	BM12-3	Foping, Shaanxi, China	N33°36.7′/E107°48.418′	1,470	ZDQ15017	MH593359
*F. yuzhongensis*	BM13-1	Yuzhong, Shaanxi, China	N35°44.160′/E103°18.870′	3,552	ZDQ14003	MH244911
*F. yuzhongensis*	BM13-2	Yuzhong, Shaanxi, China	N35°44.160′/E103°18.870′	3,552	ZDQ14003	MN810969
*F. yuzhongensis*	BM13-3	Yuzhong, Shaanxi, China	N35°44.160′/E103°18.870′	3,552	ZDQ14003	MN810970
*F. sinica*	BM14-1	Luding, Sichuan, China	N29°32.860′/101°58.250′	3,900	ZDQ15023	MH244912
*F. sinica*	BM14-3	Luding, Sichuan, China	N29°32.860′/101°58.250′	3,900	ZDQ15023	MN810971
*F. dajinensis*	BM15-1	Jinchuan, Sichuan, China	N31°09.680′/102°06.700′	4,129	ZDQ15021	MH244913
*F. dajinensis*	BM15-2	Jinchuan, Sichuan, China	N31°09.680′/102°06.700′	4,129	ZDQ15021	MN810972
*F. dajinensis*	BM15-3	Jinchuan, Sichuan, China	N31°09.680′/102°06.700′	4,129	ZDQ15021	MN810973
*F. thunbergii*	BM16-1	Dongyang, Zhejiang, China	N29°01.180′/E120°20.830′	230	ZDQ15009	MH244914
*F. thunbergii*	BM16-2	Dongyang, Zhejiang, China	N29°01.180′/E120°20.830′	230	ZDQ15009	MH593360
*F. thunbergii*	BM17-1	Nantong, Jiangsu, China	N31°55.770′/E121°00.230′	5	ZDQ16017	MH593361
*F. thunbergii*	BM17-2	Nantong, Jiangsu, China	N31°55.770′/E121°00.230′	5	ZDQ16017	MH593362
*F. monantha*	BM18-1	Lin’an, Zhejiang, China	N30°10.030′/E119°13.250′	122	ZDQ15010	MN810974
*F. monantha*	BM18-2	Lin’an, Zhejiang, China	N30°10.030′/E119°13.250′	122	ZDQ15010	MN810975
*F. monantha*	BM18-3	Lin’an, Zhejiang, China	N30°10.030′/E119°13.250′	122	ZDQ15010	MN810976
*F. anhuiensis*	BM19-1	Xuancheng, Anhui, China	N30°50.560′/E118°44.760′	135	ZDQ15012	MN810977
*F. anhuiensis*	BM19-2	Xuancheng, Anhui, China	N30°50.560′/E118°44.760′	135	ZDQ15012	MN810978
*F. anhuiensis*	BM19-3	Xuancheng, Anhui, China	N30°50.560′/E118°44.760′	135	ZDQ15012	MN810979
*F. anhuiensis*	BM20-1	Guangde, Anhui, China	N30°56.383′/E119°14.817′	16	ZDQ15011	MN810980
*F. anhuiensis*	BM20-2	Guangde, Anhui, China	N30°56.383′/E119°14.817′	16	ZDQ15011	MH593363
*F. anhuiensis*	BM20-3	Guangde, Anhui, China	N30°56.383′/E119°14.817′	16	ZDQ15011	MN810981
*F. davidii*	BM21-1	Tianquan, Sichuan, China	N29°52.400′/E102°18.430′	2,218	ZDQ16001	MN810982
*F. davidii*	BM21-2	Tianquan, Sichuan, China	N29°52.400′/E102°18.430′	2,218	ZDQ16001	MN810983
*F. davidii*	BM21-3	Tianquan, Sichuan, China	N29°52.400′/E102°18.430′	2,218	ZDQ16001	MN810984
*F. tortifolia*	BM22-1	Yumin, Xinjiang, China	N45°49.400′/E82°35.020′	1,972	ZDQ16004	MN810985
*F. tortifolia*	BM22-2	Yumin, Xinjiang, China	N45°49.400′/E82°35.020′	1,972	ZDQ16004	MN810986
*F. tortifolia*	BM22-3	Yumin, Xinjiang, China	N45°49.400′/E82°35.020′	1,972	ZDQ16004	MN810987
*F. pallidiflora*	BM23-1	Gongliu, Xinjiang, China	N43°12.920′/E82°36.280′	1,178	ZDQ16011	MH593364
*F. pallidiflora*	BM23-2	Gongliu, Xinjiang, China	N43°12.920′/E82°36.280′	1,178	ZDQ16011	MH593365
*F. pallidiflora*	BM23-3	Gongliu, Xinjiang, China	N43°12.920′/E82°36.280′	1,178	ZDQ16011	MH593366
*F. walujewii*	BM24-1	Guangliu, Xinjiang, China	N43°12.920′/E82°36.280′	1,178	ZDQ16012	MN810988
*F. walujewii*	BM24-2	Guangliu, Xinjiang, China	N43°12.920′/E82°36.280′	1,178	ZDQ16012	MN810989
*F. walujewii*	BM25-1	Hejing, Xinjiang, China	N43°14.600′/E84°40.150′	2,217	ZDQ16013	MN810990
*F. walujewii*	BM25-2	Hejing, Xinjiang, China	N43°14.600′/E84°40.150′	2,217	ZDQ16013	MN810991
*F. ussuriensis*	BM26-1	Hengyuan, Liaoning, China	N41°20.460′/E125°17.060′	275	ZDQ16015	MH593367
*F. ussuriensis*	BM26-2	Hengyuan, Liaoning, China	N41°20.460′/E125°17.060′	275	ZDQ16015	MH593368
*F. ussuriensis*	BM26-3	Hengyuan, Liaoning, China	N41°20.460′/E125°17.060′	275	ZDQ16015	MH593369
*F. maximowiczii*	BM27-1	Tahe, Heilongjiang, China	N52°19.610′/E124°26.930′	393	ZDQ16016	MN810992
*F. maximowiczii*	BM27-2	Tahe, Heilongjiang, China	N52°19.610′/E124°26.930′	393	ZDQ16016	MN810993
*F. maximowiczii*	BM27-3	Tahe, Heilongjiang, China	N52°19.610′/E124°26.930′	393	ZDQ16016	MN810994
“*F. omeiensis*”	BM28-1	Emeishan, Sichuan, China	N29°30.520′/E103°19.860′	3,026	ZDQ16003	MN810995
“*F. omeiensis*”	BM28-2	Emeishan, Sichuan, China	N29°30.520′/E103°19.860′	3,026	ZDQ16003	MN810996
“*F. omeiensis*”	BM28-3	Emeishan, Sichuan, China	N29°30.520′/E103°19.860′	3,026	ZDQ16003	MN810997
*F. crassicaulis*	BM29-1	Lijiang, Yunnan, China	N27°03.080′/E100°11.630′	3,766	ZDQ17001	MN810998
*F. crassicaulis*	BM29-2	Lijiang, Yunnan, China	N27°03.080′/E100°11.630′	3,766	ZDQ17001	MN810999
*F. crassicaulis*	BM29-3	Lijiang, Yunnan, China	N27°03.080′/E100°11.630′	3,766	ZDQ17001	MN811100
“*F. hupehensis*”	BM30-1	Wanzhou, Chongqing, China	N30°36.250′/E108°46.910′	1,282	ZDQ19006	MN811101
“*F. hupehensis*”	BM30-2	Wanzhou, Chongqing, China	N30°36.250′/E108°46.910′	1,282	ZDQ19006	MN811102
“*F. hupehensis*”	BM30-3	Wanzhou, Chongqing, China	N30°36.250′/E108°46.910′	1,282	ZDQ19006	MN811103

### DNA Extraction, Sequencing, and Assembly

Total genomic DNA was extracted from about 100 mg of dried leaf material according to a modified CTAB method (Doyle, 1987; Yang et al., 2014). Quantification of DNA was checked by electrophoresis on 1.2% agarose gels, and its concentration was detected using a SmartSpec™ Plus Spectrophotometer (Bio-Rad, Hercules, CA, United States). The purified total DNA (about 5 ug) was sheared by the sonication into fragments with an average length of 500 bp for constructing a paired-end library. Illumina libraries were prepared according to the manufacturer’s protocol. Then, the Illumina HiSeq 2000 system was adopted to perform paired-end sequencing at Beijing Genomics Institute (BGI, Shenzhen, China).

Raw data was filtered using Trimmomatic v.0.32 (Bolger et al., 2014) with default settings. Then, paired-end reads of clean data were filtered and assembled into contigs using GetOrganelle.py (Jin et al., 2020) with reference (*F. cirrhosa*, accession number: KF769143), calling the bowtie2 v.2.3.4.3 (Langmead and Salzberg, 2012), Blastn v.2.8.0 (Camacho et al., 2009), and SPAdes v.3.10 (Bankevich et al., 2012). The *de novo* assembly graphs were visualized and edited using Bandage v.8.0 (Wick et al., 2015), then a complete chloroplast genome was generated.

### Annotation and Sequence Submission

The plastomes were annotated by aligning to the reference sequence (KF769143) using MAFFT (Katoh and Standley, 2013) with default parameters, coupled with manual adjustment using Geneious v.11.1.4 (Kearse et al., 2012). Circular genome visualization was generated with OGDRAW v.1.3 (Lohse et al., 2013). Finally, the annotated chloroplast genomes of the 21 *Fritillaria* species were submitted to the NCBI database (Table 1).

### Variable Site Analysis

After using MAFFT v.7.129 to align the chloroplast genome sequences, Geneious software was used to adjust the sequences manually (Kearse et al., 2012; Katoh and Standley, 2013). A sliding window analysis was conducted for nucleotide variability (Pi) of the whole chloroplast genome using DnaSP v.6.11. Step size was set to be 200 bp, with a 600 bp window length (Rozas et al., 2017). Moreover, the software was adopted to calculate insertions/deletions (indels) and nucleotide variability (Pi) of all aligned datasets. P-distance, GC content, variable sites, and parsimony information sites were analyzed by MEGA v.7.0.26 (Kumar et al., 2016).

### Species Discrimination

Complete chloroplast genomes, used as DNA super-barcode, were adopted to be tested on species discrimination for the species in *Fritillaria* in China. In order to screen out suitable specific plastid regions, we further extracted three types of datasets, namely genes, intergenic spacers (IGSs), and high variable regions (HVRs) from the genomes manually to perform analysis of DNA barcoding. Three usual methods (Blast, Distance, and Tree-Building) were adopted to analyze the four datasets. For the Blast method, all sequences of these types of datasets were used as query sequences with an E-value < 1 × 10^–5^, and the BLAST program (Camacho et al., 2009) was used to query the reference database with each sample in turn to establish whether the closest hit was the conspecific species. Species identification was considered successful if all individuals of a species had a top matching hit of only the conspecific individuals (Ross et al., 2008). For the Distance method, all datasets were aligned by MAFFT v.7.129 (Katoh and Standley, 2013), and then used for calculating p-distances with MEGA v.7.0.26 (Kumar et al., 2016). Successful species discrimination indicated that the minimum uncorrected interspecific p-distance involving a species was larger than its maximum intraspecific distance (Li et al., 2011). For the Tree-Building method, all datasets were aligned by software MAFFT, and neighbor-joining (NJ) trees were constructed with p-distances in software MEGA. Plastomes of *Lilium brownii* (Accession: KY748296) and *L. bakerianum* (Accession: NC_035592) were used as outgroup for Tree-Building analysis. Species were regarded as a success if all individuals of one species formed a monophyletic group (Hollingsworth et al., 2010).

### Phylogenomic Analysis

Phylogenomic analysis was performed with plastomes and 10 high variable regions using Maximum Likelihood (ML), Maximum Parsimony (MP), and Bayesian Inference (BI) methods based on 82 individuals representing 25 species of the genus *Fritillaria*, including both the new and downloaded genomes here (Table 1 and Supplementary Table 1). Two species from *Lilium* L., namely *Lilium brownii* (Accession: KY748296) and *L. bakerianum* (Accession: NC_035592) were used as outgroups in the analysis. PAUP v.4.0a166 was used for the MP analysis with 1000 bootstrap replicates (Swofford, 2002). For the BI and ML analysis, the best substitution model was tested based on Akaike information criterion (AIC) by jModelTest v.2.1.7 (Darriba et al., 2012). The best-fitting models in these analyses were listed in Supplementary Table 2. The ML analysis was performed with RAxML v.8.2.4 (Stamatakis, 2014). In addition, 1000 replications were adopted to calculate the local bootstrap probability of each branch. The BI analysis was conducted in MrBayes v.3.2.6 (Ronquist et al., 2012). The Markov Chain Monte Carlo (MCMC) algorithm was calculated for 1,000,000 generations with a sampling tree every 1,000 generations. The first 25% of generations was discarded as burn-in. Stationary was reached when the average standard deviation of split frequencies was <0.01 and a consensus tree was constructed using the remaining trees.

### Divergence Time Estimation

Divergence times were estimated using a Bayesian method implemented in BEAST v.1.10.4 (Suchard et al., 2018). The ML tree was used as the starting tree and BEAUti (within BEAST) was used to set criteria for the analysis. GTR + G + I nucleotide-substitution model was adopted and an uncorrelated lognormal relaxed clock model with the Yule prior set was used to estimate divergence times. There are no well-documented fossils in Liliaceae currently. Therefore, four estimated calibration points were used to determine specific node priors based on the previous reports in Huang et al. (2018): (1) The crown node of *Lilium* was set to 16.84 Mya, using a normal prior distribution with standard deviation (SD) 2.93; (2) the crown node of *Fritillaria* was set to 18.12 Mya (normal prior distribution, SD 2.84); (3) the subgenus *Davidii* stem was constrained to 15.63 Mya (normal prior distribution, SD 2.73); (4) the subgenus *Fritillaria* B (basically Chinese species) crown node was included following a normal distribution with mean 8.28 Mya (SD 1.94). Two runs of 200 million generations of MCMC chains were produced for confirming convergence, sampling every 1000 generations, following a burn-in of the initial 10% of cycles. Samples were combined by LogCombiner v.1.10.4, and convergence of chains was checked in Tracer v1.7.1 (Rambaut et al., 2018) to confirm that the effective sample sizes (ESS) were greater than 200. Maximum clade credibility (MCC) trees were generated in TreeAnnotator v1.10.4 showing mean divergence time estimates with 95% HPD intervals and the MCC chronogram was visualized using software FigTree v1.4.3^1^.

## Results

### Chloroplast Genome Features of *Fritillaria*

Illumina sequencing system generated 2,461,376 to 29,801,350 paired-end reads with an average read length of 150 bp for 73 individuals on behalf of 21 *Fritillaria* species (including the suspicious species) in China. The produced 73 complete chloroplast genomes consisted of circular double-stranded DNA, ranging from 151,012 bp in *F. unibracteata* (Accession number: MH593351) to 152,888 bp in *F. davidii* (Accession number: MH593366) and shared typical quadripartite structure which consisted of a pair of IRs (26,071–26,746 bp) separated by the LSC (80,988–82,453 bp) and SSC (17,038–17,565 bp) regions (Supplementary Table 3 and Supplementary Figure 2). Overall GC content of the complete plastid genomes was 36.9%–37.1% (Supplementary Table 3). They consistently contained 115 genes, with 78 protein-coding genes, 30 tRNA genes, and 4 ribosomal RNA genes as well as *infA* (translation initiation factor gene), hypothetical ORF *ycf15* and *ycf68* (Supplementary Tables 3, 4). In general, genome features of the 21 species were quite similar in terms of their gene content, gene order, introns, intergenic spacers, and GC content (Supplementary Figure 2). All genome sequences had been submitted and deposited in GenBank. All the accession numbers are listed in Table 1.

### Sequence Variations

**FIGURE 1:**
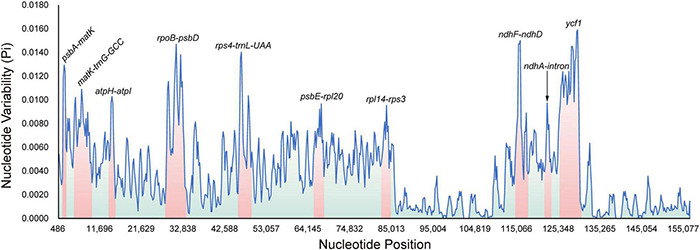
Sliding window analysis of the 73 complete plastomes in *Fritillaria* (window length: 600 bp, step size: 200 bp) to show high variable regions (HVRs). *X*-axis: position of the midpoint of a window; *Y*-axis: nucleotide diversity of each window. The size of these regions is highlighted with the obviously gradient colors.

To explore suitable plastid regions with high-resolution on species identification for *Fritillaria*, four types of datasets including chloroplast genomes, genes, intergenic spacers (IGSs), and high variable regions (HVRs) were adopted to be further analyzed in this study. A sliding window analysis found all highly divergent fragments in SC regions whereas none was presented in the IR regions. Moreover, 10 HVRs (*atpH*-*atpI*, *matK*-*trnG-GCC*, *ndhF*-*ndhD*, *psbA*-*matK*, *psbE*-*rpl20*, *rpl14*-*rps3*, *rpoB*-*psbD*, *rps4*-*trnL-UAA*, *ycf1*, and *ndhA*-intron) were selected and extracted manually from complete chloroplast genomes (Figure 1). Accordingly, 114 genes and 108 IGSs were also extracted manually from the genomes for the following analysis.

In all these datasets, undoubtedly, chloroplast genomes had the highest number of variable sites (5,940), as well as parsimony information sites (4,850) (Table 2). For HVRs, the three tops of corresponding variants were *rpoB*-*psbD* (463 and 377), *matK*-*trnG-GCC* (390 and 296), and *ycf1* (359 and 352), showing that they possessed extremely high mutations in the genome (Table 2). In genes or IGSs, there were no remarkable regions compared with HVRs; thereby, some usual fragments, such as *trnK-UUU* (150 and 149), *rpoC2* (113 and 103), and *ndhF* (111 and 104) in the genes or *trnT-UGU*-*trnL-UAA* (97 and 96), *rpl32*-*trnL-UAG* (96 and 95), and *psaJ*-*rpl33* (91 and 57) in the IGSs, exhibited fewer variation (Supplementary Table 5). Furthermore, among three datasets (HVRs, genes, and IGSs), the average intra- and inter-specific distances were positively correlated (Supplementary Figure 3). By comparative analysis of each dataset, we found that IGSs exhibited the highest interspecific variation, followed by HVRs and genes, whereas plastomes had a relatively lower variation (Supplementary Table 5 and Supplementary Figure 3).

**TABLE 2:** Characteristics of the complete plastome and high variable regions in *Fritillaria*.

	Aligned length (bp)	No. variable sites (divergence%)	No. parsimony information sites (divergence%)	No. InDels (divergence%)	Intraspecific distance		Interspecific distance		Nucleotide diversity (Pi)
					Range	Mean	Range	Mean	
Genome	157,507	5,940 (3.77%)	4,850 (3.08%)	6,384 (4.05%)	0%–0.2%	0.03%	0.02%–0.94%	0.41%	0.00416
*atpH-atpI*	978	93 (9.51%)	78 (7.98%)	130 (13.29%)	0%–0.84%	0.10%	0%–4.20%	1.35%	0.01384
*matK-trnG-GCC*	5,592	390 (6.97%)	296 (5.29%)	413 (7.39%)	0%–0.36%	0.05%	0%–2.24%	0.77%	0.00805
*ndhA-intron*	1,093	75 (6.86%)	59 (5.40%)	61 (5.58%)	0%–0.49%	0.04%	0%–2.56%	0.89%	0.00923
*ndhF-ndhD*	2,765	281 (10.16%)	188 (6.80%)	162 (5.86%)	0%–0.45%	0.04%	0%–2.92%	0.99%	0.01011
*psbA-matK*	615	71 (11.54%)	59 (9.59%)	66 (10.73%)	0%–3.75%	0.30%	0%–6.37%	2.24%	0.02239
*psbE-rpl20*	4,179	288 (6.89%)	224 (5.36%)	213 (5.10%)	0%–0.40%	0.04%	0%–1.59%	0.68%	0.00693
*rpl14-rps3*	1,788	132 (7.38%)	108 (6.04%)	145 (8.11%)	0%–0.38%	0.06%	0.06%–2.20%	0.78%	0.00801
*rpoB-psbD*	5,746	463 (8.06%)	377 (6.56%)	698 (12.15%)	0%–0.52%	0.08%	0%–2.70%	1.02%	0.00990
*rps4-trnL-UAA*	1,386	124 (8.95%)	108 (7.79%)	258 (18.61%)	0%–0.79%	0.10%	0%–3.47%	1.21%	0.01191
*ycf1*	5,586	359 (6.43%)	352 (6.30%)	96 (1.72%)	0%–0.44%	0.07%	0.02%–2.49%	1.01%	0.00972

### Species Discrimination of *Fritillaria*

**FIGURE 2:**
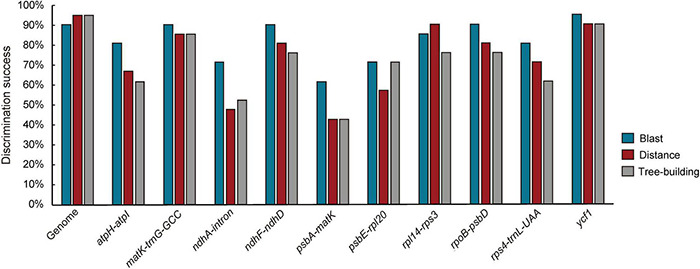
Species discrimination of the complete plastome and 10 high divergence regions (HVRs) for the 21 species in *Fritillaria* based on the three methods.

The four datasets were adopted to perform analysis on species discrimination using three methods (Blast, Distance, and Tree-Building) in this study. Among the methods, Blast tended to provide the highest success rates for most of the regions (Figure 2 and Supplementary Table 5). Moreover, it was found that all fragments without a barcoding gap between inter- and intra-specific distances could identify species (Table 2 and Supplementary Table 5). To ensure comparability of results here, the Tree-Building method (NJ) was finally adopted for discussion on species discrimination.

**FIGURE 3:**
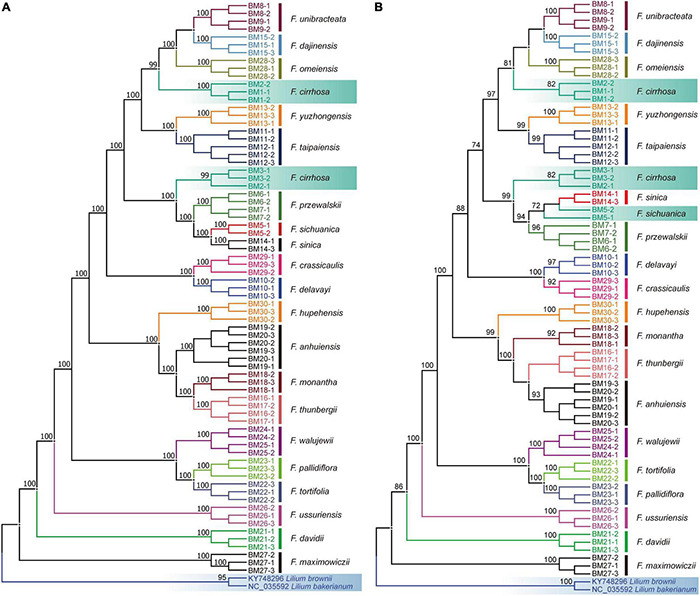
The NJ trees of the 73 *Fritillaria* samples based on the complete plastome **(A)** and *ycf1* **(B)**. Incorrect discrimination and outgroups are highlighted with the gradient colors. Bootstrap values are shown on the branches.

For complete chloroplast genome, alignment of the sequences, including two outgroups, representing 21 species in *Fritillaria* yielded a matrix containing 157,507 characters. An NJ tree was constructed based on this matrix (Figure 3A). Of note, 20 species in the genus formed a monophyletic clade with high branch supports (BS ≥ 99%). However, *F. cirrhosa* was grouped into two clades, one clade was closely related to *F. omeiensis* and the other was sister to *F. przewalskii* and *F. sichuanica* or *F. sinica*.

Compared to genes and IGSs, HVRs showed the highest success rate with good performance (Figure 2 and Supplementary Table 5). Among the plastid loci, *ycf1* exhibited great discrimination ability with high branch supports (BS ≥ 92%) contrasted with other regions (Figure 3B and Supplementary Figure 4). Only two species could not be correctly discriminated, namely *F. cirrhosa* and *F. sichuanica*. Furthermore, the *rpoC1* in these genes also showed excellent power which could identify 18 of 21 species except three closely related species, including *F. cirrhosa*, *F. dajinensis*, and *F. omeiensis* (Supplementary Figure 5). However, in the IGSs, there was no remarkable region that could significantly improve the species resolution, and the best region *rpl20*-*rps12* could only discriminate 13/21 species in *Fritillaria* (Supplementary Figure 4).

### Phylogenomic Analysis and Divergence Time Estimation

**FIGURE 4:**
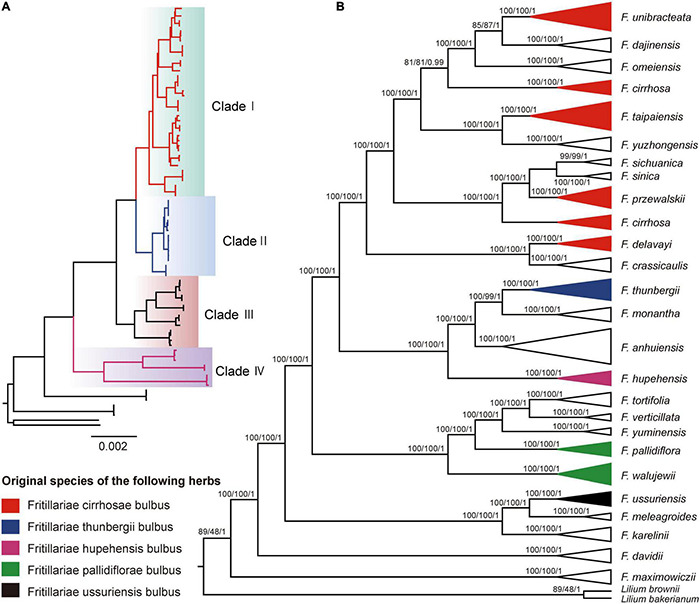
Phylogenetic analysis of 25 *Fritillaria* species inferred from maximum likelihood (ML), maximum parsimony (MP), and Bayesian analyses (BI) based on the complete chloroplast genomes. ML topological structure is shown on the left of the figure **(A)**. For the topological structure on the right of the figure **(B)**, the numbers above nodes are support values with ML bootstrap values on the left, MP bootstrap values in the middle, and Bayesian posterior probability (PP) values on the right. Colors represent the four major clades of the genus *Fritillaria* in China **(A)** and the original species of five Fritillariae bulbus of traditional Chinese medicine **(B)**.

Phylogenomic analysis was performed using ML, MP, and BI methods on the basis of 84 individuals (including two outgroups). As a result, none of the HVRs possessed similar topological structures of the three trees with that of the plastome due to their poorer information sites in phylogeny (Figure 4, Supplementary Figure 5, and Supplementary Table 2), and the support values of their branch sites were weaker in contrast with that of the whole chloroplast genome. Herein, the phylogenetic trees reconstructed by plastomes were adopted to discuss the phylogenetic relationships of *Fritillaria* in China, and the ML tree was adopted to present phylogenetic relationships, with the addition of support values from MP and BI analyses (Figure 4). According to the trees, the 23 species of *Fritillaria* sect. *Fritillaria*, except *F. davidii* and *F. maximowiczii*, were obviously divided into four major clades (Clade I, II, III, and IV). Clade I contained 11 species with strong supports (BS = 100%, PP = 1.00). Within this clade, five original species of the herbs Fritillariae cirrhosae bulbus had closer relationships with each other. Besides, the species *F. cirrhosa* was grouped into two subclades with high support values, which was similar to the result of the NJ method. Within the second clade, four species, containing three widely cultivated herbs, exhibited a clear relationship in which the species *F. monantha* was sister to *F. thunbergii* with high support value (BS = 100%, PP = 1.00). For the third clade, five species, almost distributed in Xinjiang, showed an unambiguous relationship with 100% support. Among these species, *F. tortifolia* was sister to *F. verticillate* and then related to *F. yuminensis*, but the two original species of Fritillariae pallidiflorae bulbus were slightly far away from them. The last clade showed that *F. ussuriensis* was sister to *F. meleagroides* (BS = 100%, PP = 1.00).

**FIGURE 5:**
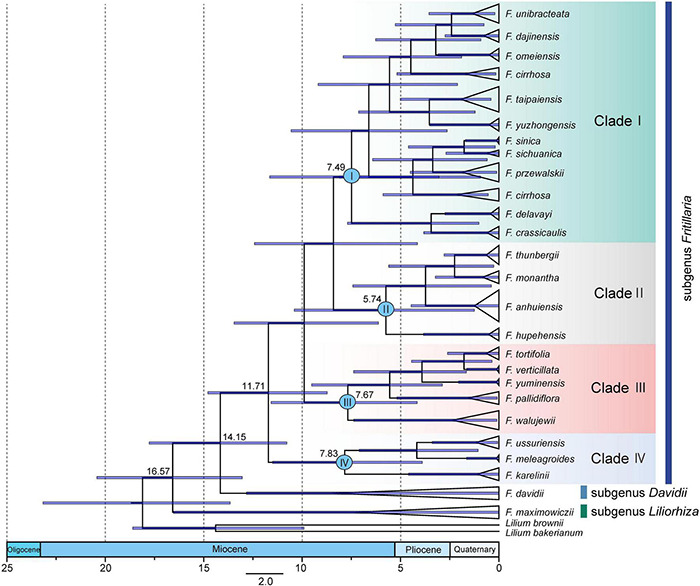
Maximum clade credibility (MCC) tree of the 25 *Fritillaria* species from BEAST analysis under the Yule tree prior and lognormal uncorrelated relaxed clock models on the basis of complete plastomes. Blue bars represent 95% credibility intervals for each node with posterior probability above 0.5.

The divergence times of these three subgenera were inferred based on BEAST chronogram using 84 plastomes (Figure 5). All the nodes in the tree were highly supported with a posterior probability of more than 0.9. The subgenus *Fritillaria* was divided into four major clades. Clade I was estimated to be 7.49 Mya (95% HPD, 3.04∼11.65 Mya). The sister group Clade II was estimated at 5.74 (1.24∼10.40) Mya, while the remaining clade of Clade III and Clade IV split at 7.67 (4.15∼11.58) Mya and 7.83 (3.89∼11.50) Mya, respectively. The speciation events of this subgenus started from 11.71 (8.73∼14.79) Mya evolved in the Middle and Late Miocene. Furthermore, the subgenus *Davidii* and *Liliorhiza* including only one species in China were split at 14.15 (10.77∼17.77) Mya and 16.57 (13.04∼20.43) Mya, respectively, which also occurred in the Miocene.

## Discussion

### Species Discrimination in *Fritillaria* Based on DNA Super-Barcodes

The *Fritillaria* is an important genus with complicated phylogeny, especially the “*Fritillaria cirrhosa* complex” in southwest China. Our previous study revealed that eight species (*F. cirrhosa*, *F. sichuanica*, *F. taipaiensis*, *F. yuzhongensis*, *F. unibracteata*, *F. przewalskii*, *F. sinica*, and *F. dajinensis*) were closely related due to their confused morphology and close phylogenetic relationships (Chen et al., 2019; Wu et al., 2020). For such a complicated group, the standard DNA barcodes, using Sanger sequencing, had been proved to be ineffective (Zhang et al., 2016); whereas complete chloroplast genome, as a super-barcode, showed powerful ability on species discrimination (Chen et al., 2020). In this study, all the 21 *Fritillaria* species in China, except *F. cirrhosa*, could be well identified based on the 73 chloroplast genomes, including two suspicious species, namely “*F. omeiensis*” and “*F. hupehensis*” (Figure 3A). These results indicated that a complete chloroplast genome could effectively improve the resolution of species identification of DNA barcoding in *Fritillaria*, compared with the universal DNA barcodes. Therefore, super-barcode could be listed as universal or complementary DNA barcode in plants, especially complicated taxa in taxonomy and phylogeny.

Complete chloroplast genomes had been proposed as the candidates for the next-generation DNA barcodes in plants (Li et al., 2015; Hollingsworth et al., 2016; Tontifilippini et al., 2017; Ji et al., 2019). Performance of cpDNA genomes on species discrimination had been tested from a series of genera (Nock et al., 2011; Kane et al., 2012; Ruhsam et al., 2015; Krawczyk et al., 2018; Fu et al., 2019; Ji et al., 2019; Kreuzer et al., 2019; Yang et al., 2019). The genomes could perform well in complicated *Taxus* (Fu et al., 2019), but they were also faced with difficulty on identifying species in *Panax*, *Araucaria*, *Notopterygium*, and *Berberis* (Ruhsam et al., 2015; Ji et al., 2019; Kreuzer et al., 2019; Yang et al., 2019). Herein, “*Fritillaria cirrhosa* complex,” as a group of recent diversification and rapid evolution (Luo and Chen, 1996a; Xiao et al., 2007), possesses extremely complicated morphological variations (Supplementary Figure 1) and phylogenetic relationships (Chen et al., 2019, 2020; Wu et al., 2020), which results in difficulty and dispute in taxonomy. Thus, complete plastome provided rich genetic variants and strong ability on species discrimination here, but it could not effectively track the boundary of *F. cirrhosa* which was also emphasized in previous research (Hollingsworth, 2011; Li et al., 2015). Therefore, it should be noticed that super-barcode was still not omnipotent, DNA barcodes from different genetic systems are required (Ruhsam et al., 2015; Hollingsworth et al., 2016; Ji et al., 2019).

### Screening Specific Regions for Discriminating *Fritillaria* Species

**FIGURE 6:**
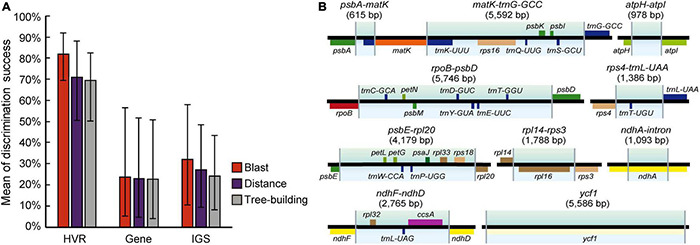
Average discriminatory power with error bars donated standard deviation (SD) of HVRs, genes, and IGSs based on three methods **(A)** and the aligned length of HVRs and genes/IGSs involved in them **(B)**. The size of these regions is highlighted with the gradient colors. Genes above the line are transcribed clockwise, and genes shown over the line are counterclockwise. Rectangles in assorted colors represent the corresponding gene function. More details about these functions are shown in Supplementary Figure 2 and Supplementary Table 4.

The goal of DNA barcoding in plants is to increase success rates of species identification in which unique results of species identifications could be achieved (CBOL Plant Working Group, 2009). It was reported that lineage-specific barcodes could enhance resolution of the discrimination within a particular group owing to richer genetic information than the universal barcodes (Li et al., 2015). In this study, the HVRs could gain higher discriminatory resolution than that of genes and IGSs (Figures 2, 6A and Supplementary Table 5), demonstrating that the HVRs were potential fragments that could obviously increase the resolution for identifying *Fritillaria* species, and a similar result was also reported in *Pterocarpus* (Jiao et al., 2019). Furthermore, it should be noted that some HVRs possessed much longer lengths, including one or more adjacent genes and IGSs (Figure 6B). Thus, development on longer length of DNA sequencing would be beneficial to adopt specific DNA barcodes in the complicated group.

A limited number of fragments selected from plastomes were tested and got a good performance in species identification in recent studies, such as *ndhF-rpl32* or *psbE*-*psbL*/*ndhA* intron for *Fagopyrum* (Hu et al., 2016; Huang et al., 2019), *rpl32*-*ccsA* for *Dioscorea* (Scarcelli et al., 2011), and *trnL-trnF* or *accD*/*rrn16*-*rrn23* for yew species (Liu et al., 2018; Fu et al., 2019). However, in *Fritillaria*, the presented study indicated that all these special loci could not afford higher discriminatory power than that of the universal DNA barcodes (*matK* + *rbcL*) (Supplementary Table 5). In contrast, three plastid loci (*ycf1*, *matK-trnG-GCC*, and *rpoC1*) showed better resolution for identifying the species in *Fritillaria*. Among these regions, *ycf1* has been proposed as the most potential DNA barcode from chloroplast genome for land plants (Dong et al., 2015), but *rpoC1* provided low resolution in many groups due to short DNA sequences (CBOL Plant Working Group, 2009; Wyler and Naciri, 2016; Xie et al., 2019). Moreover, *matK-trnG-GCC* included more variable sites (Table 2). Consequently, these three loci were suitable and could be proposed as the specific DNA barcodes from cpDNA genome for identifying *Fritillaria* species. Nevertheless, according to the selection criteria of DNA barcodes (Pečnikar and Buzan, 2013), the candidate loci can be excluded for specific DNA barcodes due to their length because this case might increase difficulty in designing primers, amplification, and sequencing. For the remaining loci, only the *matK* can provide high discriminatory success in *Fritillaria* species (Supplementary Table 5), which was also supported by our previous reports (Chen et al., 2020). Therefore, the universal barcode could also be regarded as the special DNA barcode in *Fritillaria* to some extent.

### Phylogenetic Relationships and Divergence Time of *Fritillaria* Species in China

**FIGURE 7:**
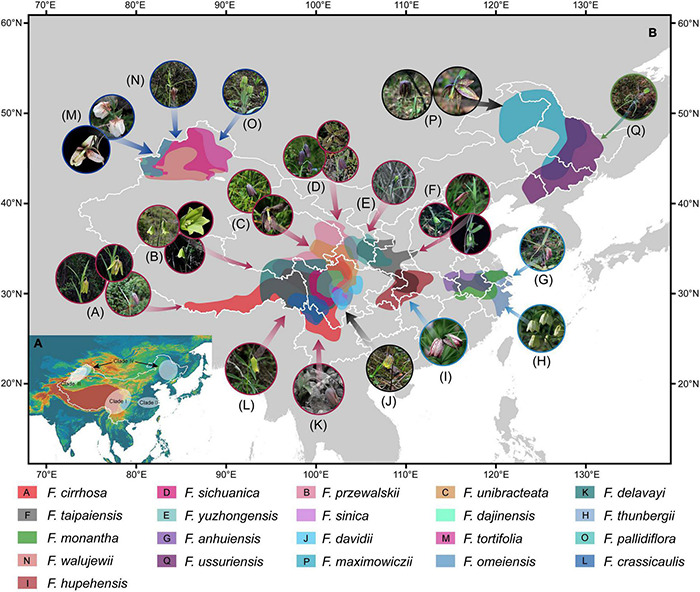
Four major clades of the subgenus *Fritillaria* in geography **(A)** and the potential geographical distributions of the 21 *Fritillaria* species predicted using the MaxEnt model with all records of existing vouchers of corresponding species (http://www.cvh.ac.cn/ and http://www.papc.cn/) **(B)**. Photos represent living plants of *Fritillaria* species as follows: (A) *F. cirrhosa*, (B) *F. przewalskii*, (C) *F. unibracteata*, (D) *F. sichuanica*, (E) *F. yuzhongensis*, (F) *F. taipaiensis*, (G) *F. anhuiensis*, (H) *F. thunbergii*, (I) “*F. hupehensis*,” (J) *F. davidii*, (K) *F. delavayi*, (L) *F. crassicaulis*, (M) *F. tortifolia*, (N) *F. walujewii*, (O) *F. pallidiflora*, (P) *F. maximowiczii*, and (Q) *F. ussuriensis*.

Recently, the complete chloroplast genome has been verified as a useful tool that could enhance phylogenetic resolution for complicated genera in angiosperm on account of its abundant informative sites (Wu et al., 2010; Xue et al., 2012; Bayly et al., 2013; Carbonell-caballero et al., 2015; Gitzendanner et al., 2017). In this study, 25 species in *Fritillaria*, including 23 species recorded by *Flora of China* (2000), as well as two suspicious species, were adopted to explore the phylogeny, which almost represented most of the recognized species in China (Table 1 and Supplementary Table 1). Phylogenomic analysis supported the monophyly of the subgenus *Fritillaria.* More importantly, 23 species in the subgenus could be divided into four major clades (Clade I, II, III, and IV) with high support values (Figure 4), in accordance with the previous findings based on chloroplast regions and ITS (Day et al., 2014; Huang et al., 2018; Zhang et al., 2021). The four clades possessed obvious geographic structure except the last one (Figures 4, 7). Among them, the first clade, distributed in southwest China, was undoubtedly the most complicated group probably due to its recent diversification. This work provided a comprehensive topological structure for the *Fritillaria* in China although the previous studies have revealed similar results based on limited samples and DNA markers (Park et al., 2017; Bi et al., 2018; Huang et al., 2018; Li et al., 2018; Zhang et al., 2021). Moreover, it is worth noting that of the suspicious species, “*F. omeiensis*” was not clustered into the same clade with *F. crassicaulis*, which was treated as a synonym for the latter in *Flora of China* (2000); similarly, “*F. hupehensis*” was also far from the *F. monantha* (Figure 4). Therefore, taxonomic status of the two suspicious species in *Flora of China* (2000) might be questionable, which needs further verification according to morphological and molecular evidence.

The *F. cirrhosa* complex is an extremely complicated group distributed in the southwest of China, which might constitute more than the four species defined by professor Yibo Luo (Luo and Chen, 1996a; Xiao et al., 2007; Chen et al., 2019). In this region, distributions of the closely related species are almost adjacent or intersected with each in geographic structure (Figure 7), which may be an important reason that causes the complex relationships and similar morphological traits among the species. A previous study suggested that all species of the *F. cirrhosa* complex could be used as the sources supplying Fritillariae cirrhosae bulbus due to their similar chemical compounds in bulbs and morphological characteristics in plants (Xiao et al., 2007). Indeed, our investigation in field work also revealed that bulbs of most of the *Fritillaria* species in this region were generally used as Fritillariae cirrhosae bulbus by aboriginals for many years. Moreover, there were very close relationships among the five original species of Fritillariae cirrhosae bulbus, as well as the remaining species (Figure 4), and they were closely adjacent in geographical distribution (Figure 7). Therefore, it is feasible to broaden the original species of the medicine-based phylogeny and medicinal history, but it needs further verifications from the pharmacy and other disciplines.

Mountain barriers may have played vital roles in speciation and diversification because topographic complexity could lead to ecological stratification and environmental heterogeneity (Fjeldså et al., 2012). In this study, our results showed that the divergence time of subgenus *Fritillaria* in China dated to 11.71 Mya (8.73∼14.79 Mya, 95% HPD) in the Middle to Late Miocene period (Figure 5). The four major clades were estimated at 5∼8 Mya evolved in the Late Miocene within this subgenus. However, due to the limited representative species and outgroups in our analysis, molecular dating might not designate an exact origin time, so that these times are relatively earlier than previously published estimations (Huang et al., 2018). Furthermore, geological data and many biogeographical studies confirmed that the Qinghai-Tibetan Plateau (QTP) uplift occurred from the Miocene to the mid-Pliocene until the start of the Quaternary (Li et al., 1979; Zhou et al., 2006; Royden et al., 2008). Therefore, we speculated that the origin and divergence of *Fritillaria* species in China could be closely associated with uplift of the QTP. With continued orogeny of the Himalayas in that period, the QTP alongside was subjected to various uplift events that led to climate cooling in these regions (Jin et al., 2003). When temperatures fell, plants adapted to cold habitats might have expanded their range outside the QTP to other newly available temperate areas (Zhang et al., 2014). Thus, these dispersal events might be the important reason for the early diversification of *Fritillaria* species into its major extant Chinese clades in geographical distribution (Figure 7).

## Conclusion

In the present study, we tried to evaluate resolution of the complete plastomes in species discrimination, phylogenetic reconstruction, and divergence time estimation of major clades in *Fritillaria*. The results indicated that the whole plastomes could improve resolution in species discrimination but could not fully match the species boundaries in *Fritillaria*. Based on the comparative analysis of many fragments, we found that four regions (*ycf1*, *matK*-*trnG-GCC*, *rpoC1*, and *matK*) could gain high discriminatory power but three of them were not suitable loci in length as special DNA barcodes for identification of *Fritillaria* species except the plastid gene *matK*. Moreover, the present phylogenomic analysis was by far the most comprehensive study to reveal relationships of the *Fritillaria* species in China, which showed that the subgenus *Fritillaria* was divided into four major clades and the taxonomic delimitation of two suspicious species (“*F. omeiensis*” and “*F. hupehensis*”) by *Flora of China* (2000) might be questionable and need to be further revised. The original times of the section *Fritillaria* species were estimated, and the results illustrated that both the subgenus *Fritillaria* and its four main clades were evolved in the Middle to Late Miocene. In conclusion, the newly developed plastomes resources and comparative analysis along with the existing plastomes of *Fritillaria* would be beneficial to promoting the rational utilization of medicinal species in the important genus.

## Data Availability Statement

The datasets presented in this study can be found in online repositories. The names of the repository/repositories and accession number(s) can be found in the article/Supplementary Material.

## Author Contributions

DZ designed the study and collected molecular materials. QC and HH generated molecular data and performed data analysis. QC wrote an initial draft of the manuscript. DZ revised this manuscript finally and submitted it. All authors contributed to the article and approved the submitted version.

## Conflict of Interest

The authors declare that the research was conducted in the absence of any commercial or financial relationships that could be construed as a potential conflict of interest.

## Publisher’s Note

All claims expressed in this article are solely those of the authors and do not necessarily represent those of their affiliated organizations, or those of the publisher, the editors and the reviewers. Any product that may be evaluated in this article, or claim that may be made by its manufacturer, is not guaranteed or endorsed by the publisher.

## Footnotes

1
https://github.com/rambaut/figtree/releases/tag/v1.4.3

## Supplementary Material

Supplementary Figure 1 Plant morphology of the *Fritillaria* species in this study.

Supplementary Figure 2 Gene map of chloroplast genomes of *Fritillaria*. Genes outside the circle are transcribed clockwise, and genes shown on the inside of the circle are counterclockwise. Genes belonging to the functional group are color-coded. The darker gray in the inner corresponds to GC content, and the lighter gray corresponds to AT content.

Supplementary Figure 3 Positive correlation between intra- and inter-specific p-distances of (A) high divergence regions (HVRs), (B) genes, and (C) intergenic spacer (IGS) in the 21 *Fritillaria* species.

Supplementary Figure 4 Performances of other HVRs for identifying the *Fritillaria* species using NJ trees.

Supplementary Figure 5 Phylogenetic relationship of the 25 *Fritillaria* species inferred from maximum likelihood (ML), maximum parsimony (MP), and Bayesian analyses (BI) based on the 10 high divergence regions (HVRs). If three trees possessed similar topological structures, the ML tree was declared to present phylogenetic relationships with the addition of support values from MP and BI analyses. Or else, just put them on their own, respectively. Bootstrap values or Bayesian posterior probability (PP) values less than 60 (or 0.6) are not exhibited in the phylogenetic trees.
