# Supplementary material for: DNA Barcoding and Phylogenomic Analysis of the Genus Fritillaria in China Based on Complete Chloroplast Genomes
Source: Front Plant Sci. 2022 Feb 25;13:764255. doi: 10.3389/fpls.2022.764255 (PMC8914171; doi:10.3389/fpls.2022.764255)
Supplement: Supplementary Figure 1 — Plant morphology of the Fritillaria species in this study. [file Data_Sheet_1.zip › Figure S1.PDF]

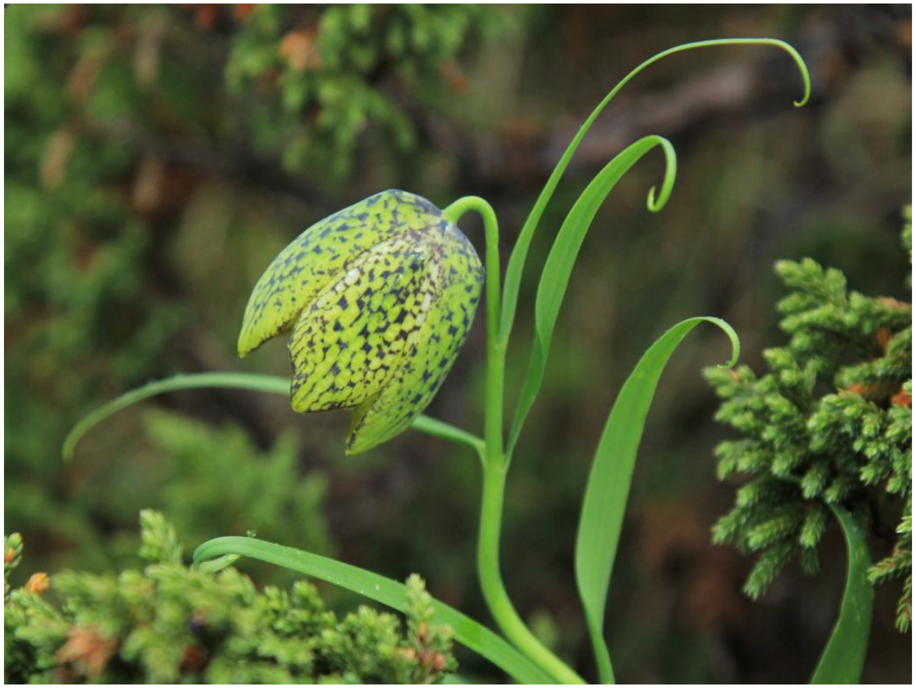

Fritillaria cirrhosa (Lijiang)

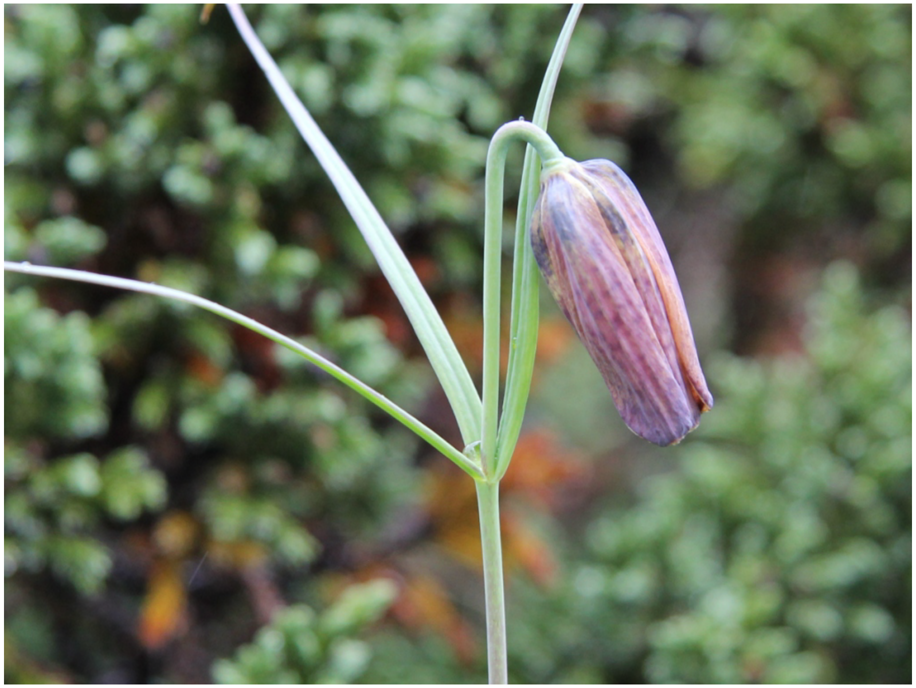

Fritillaria cirrhosa (Shangri-La)

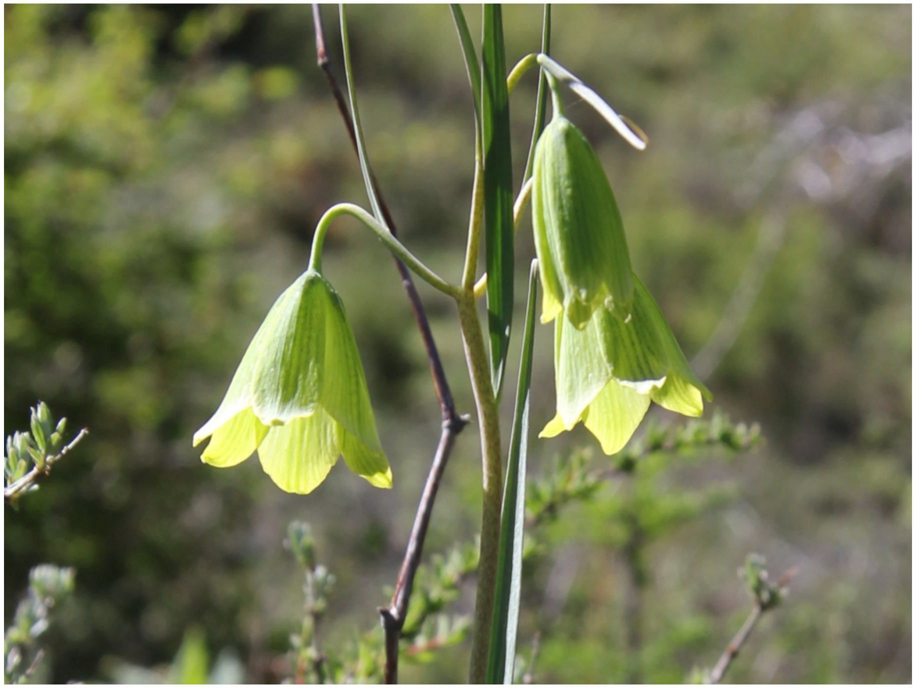

Fritillaria przewalskii (Ganzi)

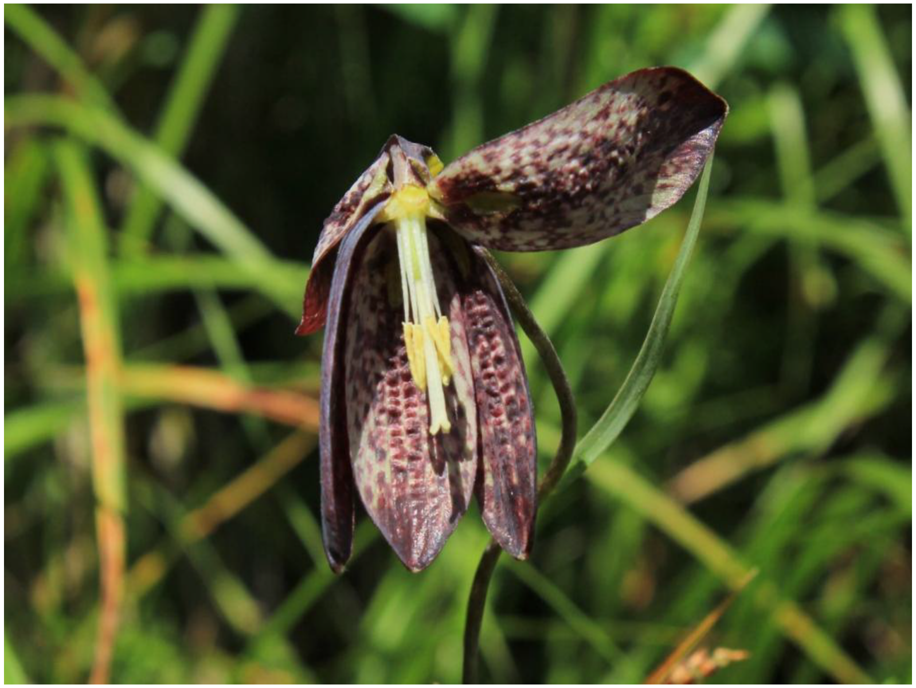

Fritillaria unibracteata (Hongyuan)

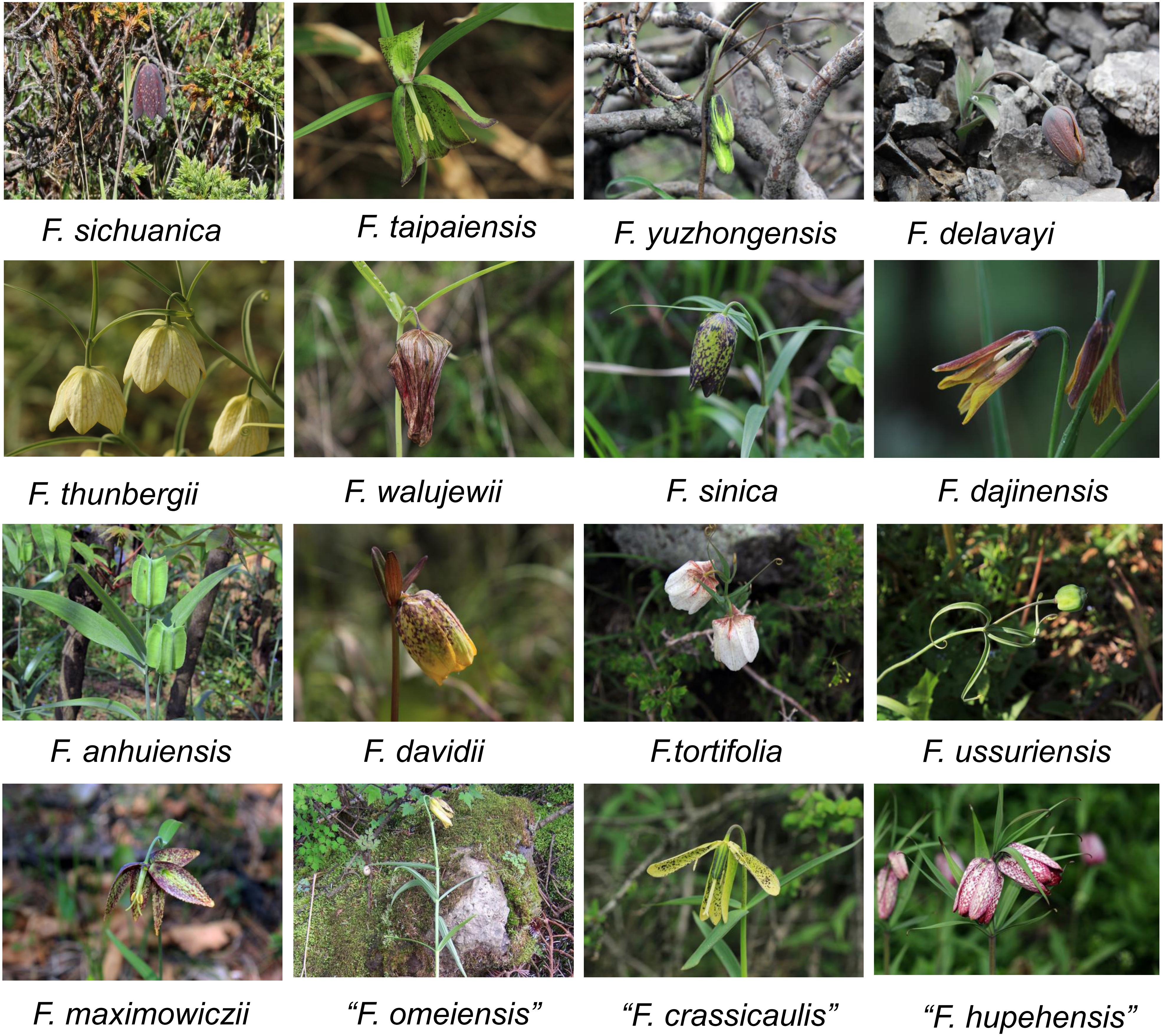
F. sichuanica
F. taipaiensis
F. yuzhongensis
F. delavayi
F. thunbergii
F. walujewii
F. sinica
F. dajinensis
F. anhuiensis
F. davidii
F.tortifolia
F. ussuriensis
F. maximowiczii
"F. omeiensis"
"F. crassicaulis"
"F. hupehensis"
