# Supplementary material for: DNA Barcoding and Phylogenomic Analysis of the Genus Fritillaria in China Based on Complete Chloroplast Genomes
Source: Front Plant Sci. 2022 Feb 25;13:764255. doi: 10.3389/fpls.2022.764255 (PMC8914171; doi:10.3389/fpls.2022.764255)
Supplement: Supplementary Figure 1 — Plant morphology of the Fritillaria species in this study. [file Data_Sheet_1.zip › Figure S2.PDF]

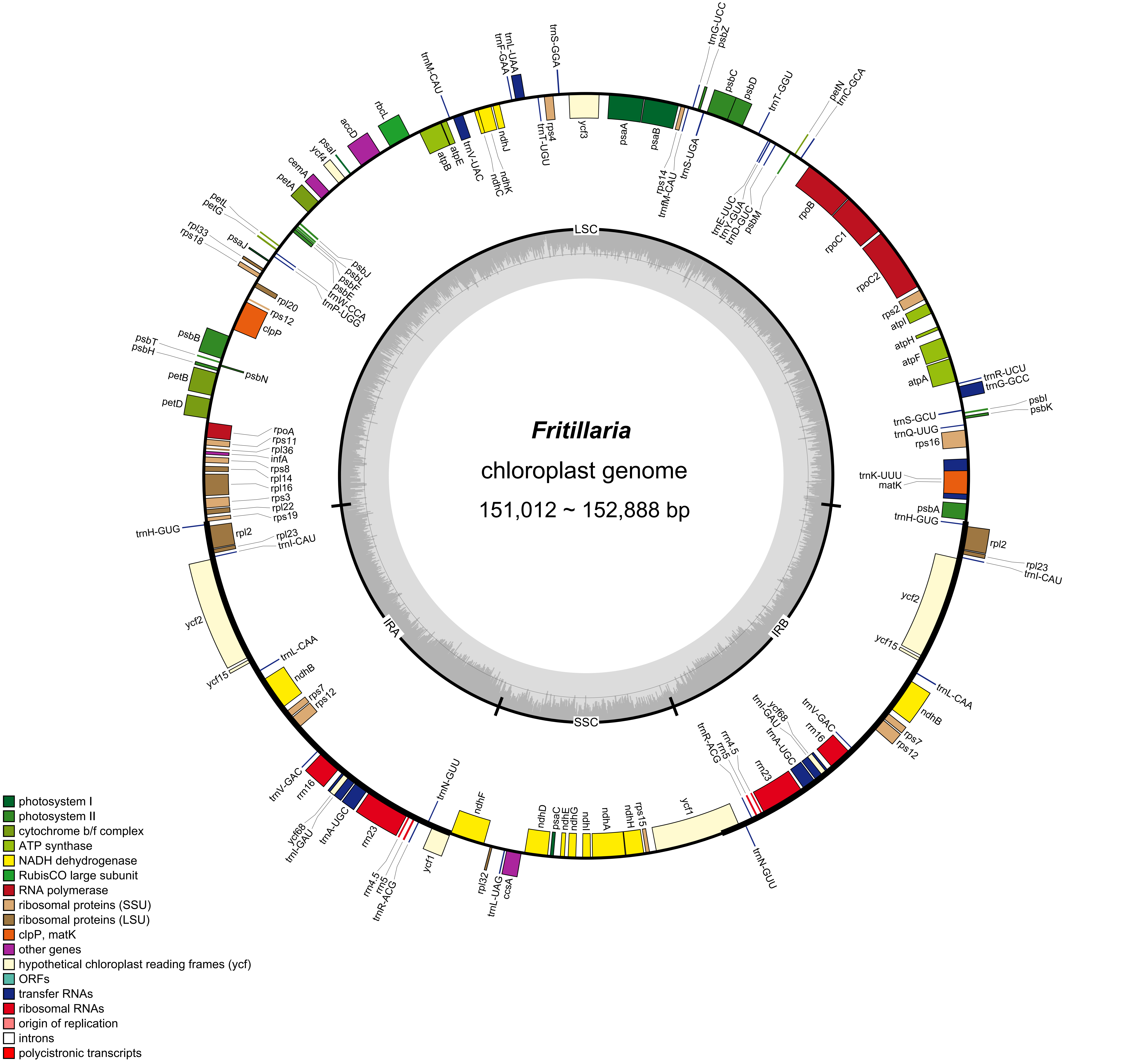
Fritillaria
chloroplast genome
151,012 ~ 152,888 bp
LSC
IRA
IRB
SSC
trnG-UCC
psbZ
psbC
psbD
trnT-GGU
petN
trnC-GCA
trnS-GGA
trnL-UAA
trnF-GAA
trnM-CAU
rbcL
accD
psaI
ycf4
cemA
petA
petL
petG
psaJ
rpl33
rps18
rpl20
rps12
clpP
psbB
psbT
psbH
psbN
petB
petD
rpoA
rps11
rpl36
infA
rps8
rpl14
rpl16
rps3
rpl22
rps19
trnH-GUG
rpl2
rpl23
trnI-CAU
ycf2
ycf15
trnL-CAA
ndhB
rps7
rps12
trnV-GAC
rrn16
ycf68
trnI-GAU
trnA-UGC
rrn23
rrn4.5
rrn5
trnR-ACG
trnN-GUU
ycf1
ndhF
rpl32
trnL-UAG
ccsA
ndhD
psaC
ndhE
ndhG
ndhI
ndhA
ndhH
rps15
ycf1
trnN-GUU
trnR-ACG
rrn4.5
rrn5
rrn23
trnA-UGC
trnI-GAU
ycf68
rrn16
trnV-GAC
trnL-CAA
ndhB
rps7
rps12
ycf15
ycf2
rpl23
trnI-CAU
rpl2
trnH-GUG
psbA
trnK-UUU
matK
rps16
trnQ-UUG
trnS-GCU
psbI
psbK
trnR-UCU
trnG-GCC
atpA
atpF
atpH
atpI
rps2
rpoC2
rpoC1
rpoB
psbM
trnD-GUC
trnY-GUA
trnE-UUC
trnS-UGA
trnfM-CAU
rps14
psaB
psaA
ycf3
rps4
trnT-UGU
ndhJ
ndhK
ndhC
trnV-UAC
atpE
atpB
psbJ
psbL
psbF
psbE
trnW-CCA
trnP-UGG
photosystem I
photosystem II
cytochrome b/f complex
ATP synthase
NADH dehydrogenase
RubisCO large subunit
RNA polymerase
ribosomal proteins (SSU)
ribosomal proteins (LSU)
clpP, matK
other genes
hypothetical chloroplast reading frames (ycf)
ORFs
transfer RNAs
ribosomal RNAs
origin of replication
introns
polycistronic transcripts
