# Supplementary material for: DNA Barcoding and Phylogenomic Analysis of the Genus Fritillaria in China Based on Complete Chloroplast Genomes
Source: Front Plant Sci. 2022 Feb 25;13:764255. doi: 10.3389/fpls.2022.764255 (PMC8914171; doi:10.3389/fpls.2022.764255)
Supplement: Supplementary Figure 1 — Plant morphology of the Fritillaria species in this study. [file Data_Sheet_1.zip › Table S1.DOCX]

**SUPPLEMENTARY TABLE 1 |** Accession numbers of plastomes downloaded from GenBank database in this study.

| Taxon | Plastome size (bp) | GenBank Accession |
| --- | --- | --- |
| *Fritillaria karelinii* | 152,118 | KX354691 |
| *Fritillaria karelinii* | 152,112 | MG211818 |
| *Fritillaria karelinii* | 152,112 | NC_037213 |
| *Fritillaria meleagroides* | 151,846 | MF947710 |
| *Fritillaria meleagroides* | 151,846 | NC_037040 |
| *Fritillaria verticillata* | 151,959 | MG211823 |
| *Fritillaria verticillata* | 151,959 | NC_037217 |
| *Fritillaria yuminensis* | 151,813 | MG200070 |
| *Fritillaria yuminensis* | 151,813 | NC_037209 |
