# Supplementary material for: DNA Barcoding and Phylogenomic Analysis of the Genus Fritillaria in China Based on Complete Chloroplast Genomes
Source: Front Plant Sci. 2022 Feb 25;13:764255. doi: 10.3389/fpls.2022.764255 (PMC8914171; doi:10.3389/fpls.2022.764255)
Supplement: Supplementary Figure 1 — Plant morphology of the Fritillaria species in this study. [file Data_Sheet_1.zip › Table S2.DOCX]

**SUPPLEMENTARY TABLE 2 |** Data characteristics and selected best fit model in each data set.

|  | Aligned length (bp) | NO. variable sites | NO. parsimony information sites | Best-fitting model |
| --- | --- | --- | --- | --- |
| Genome | 157,507 | 5,940 | 4,850 | GTR+I+G |
| *atpH-atpI* | 985 | 89 | 75 | GTR+I+G |
| *matK-trnG-GCC* | 5,631 | 320 | 258 | GTR+I+G |
| *ndhA-intron* | 1,109 | 75 | 63 | GTR+G |
| *ndhF-ndhD* | 2,804 | 203 | 182 | GTR+I+G |
| *psbA-matK* | 637 | 57 | 48 | GTR+G |
| *psbE-rpl20* | 4,204 | 206 | 172 | GTR+I+G |
| *rpl14-rps3* | 1,892 | 127 | 110 | GTR+I+G |
| *rpoB-psbD* | 5,779 | 301 | 256 | GTR+I+G |
| *rps4-trnL-UAA* | 1,404 | 80 | 72 | GTR+I+G |
| *ycf1* | 5,611 | 438 | 386 | GTR+I+G |
