# Supplementary material for: DNA Barcoding and Phylogenomic Analysis of the Genus Fritillaria in China Based on Complete Chloroplast Genomes
Source: Front Plant Sci. 2022 Feb 25;13:764255. doi: 10.3389/fpls.2022.764255 (PMC8914171; doi:10.3389/fpls.2022.764255)
Supplement: Supplementary Figure 1 — Plant morphology of the Fritillaria species in this study. [file Data_Sheet_1.zip › Table S3.DOCX]

**SUPPLEMENTARY TABLE 3 |** Summary of complete chloroplast genomes for the 21 *Fritillaria* species.

| Species | Number | Total (bp) | Large single copy  (LSC, bp) | Small single copy  (SSC, bp) | Inverted repeat  (IR, bp) | Total genes | Protein coding genes | tRNA | rRNA | GC% | Accession number |
| --- | --- | --- | --- | --- | --- | --- | --- | --- | --- | --- | --- |
| *F. cirrhosa* | BM1-1 | 151,546 | 81,402 | 17,542 | 26,301 | 115 | 78 | 30 | 4 | 37.0% | MH593342 |
|  | BM1-2 | 151,546 | 81,402 | 17,542 | 26,301 | 115 | 78 | 30 | 4 | 37.0% | MH593343 |
|  | BM2-1 | 151,998 | 81,755 | 17,545 | 26,349 | 115 | 78 | 30 | 4 | 37.0% | MH244906 |
|  | BM2-2 | 151,605 | 81,467 | 17,534 | 26,302 | 115 | 78 | 30 | 4 | 37.0% | MH593344 |
|  | BM3-1 | 152,035 | 81,794 | 17,541 | 26,350 | 115 | 78 | 30 | 4 | 36.9% | MH593345 |
|  | BM3-2 | 152,035 | 81,794 | 17,541 | 26,350 | 115 | 78 | 30 | 4 | 36.9% | MH593346 |
| *F. sichuanica* | BM5-1 | 152,062 | 81,823 | 17,539 | 26,350 | 115 | 78 | 30 | 4 | 36.9% | MN810967 |
|  | BM5-2 | 152,061 | 81,820 | 17,541 | 26,350 | 115 | 78 | 30 | 4 | 36.9% | MN810968 |
| *F. przewalskii* | BM6-1 | 151,983 | 81,744 | 17,539 | 26,350 | 115 | 78 | 30 | 4 | 36.9% | MH244908 |
|  | BM6-2 | 152,054 | 81,816 | 17,538 | 26,350 | 115 | 78 | 30 | 4 | 36.9% | MH593347 |
|  | BM7-1 | 151,955 | 81,715 | 17,540 | 26,350 | 115 | 78 | 30 | 4 | 37.0% | MH593348 |
|  | BM7-2 | 151,960 | 81,722 | 17,538 | 26,350 | 115 | 78 | 30 | 4 | 37.0% | MH593349 |
| *F. unibracteata* | BM8-1 | 151,058 | 81,339 | 17,539 | 26,090 | 115 | 78 | 30 | 4 | 37.0% | MH244909 |
|  | BM8-2 | 151,057 | 81,338 | 17,539 | 26,090 | 115 | 78 | 30 | 4 | 37.0% | MH593350 |
|  | BM9-1 | 151,012 | 81,295 | 17,537 | 26,090 | 115 | 78 | 30 | 4 | 37.0% | MH593351 |
|  | BM 9-2 | 151,078 | 81,398 | 17,538 | 26,071 | 115 | 78 | 30 | 4 | 37.0% | MH593352 |
| *F. delavayi* | BM10-1 | 151,853 | 81,602 | 17,513 | 26,369 | 115 | 78 | 30 | 4 | 37.0% | MH593353 |
|  | BM10-2 | 151,854 | 81,603 | 17,513 | 26,369 | 115 | 78 | 30 | 4 | 37.0% | MH593354 |
|  | BM10-3 | 151,854 | 81,603 | 17,513 | 26,369 | 115 | 78 | 30 | 4 | 37.0% | MH593355 |
| *F. taipaiensis* | BM11-1 | 151,707 | 81,451 | 17,552 | 26,352 | 115 | 78 | 30 | 4 | 37.0% | MH244910 |
|  | BM11-2 | 151,518 | 81,268 | 17,546 | 26,352 | 115 | 78 | 30 | 4 | 37.0% | MH593356 |
|  | BM12-1 | 151,741 | 81,478 | 17,561 | 26,351 | 115 | 78 | 30 | 4 | 37.0% | MH593357 |
|  | BM12-2 | 151,741 | 81,478 | 17,561 | 26,351 | 115 | 78 | 30 | 4 | 37.0% | MH593358 |
|  | BM12-3 | 151,741 | 81,478 | 17,561 | 26,351 | 115 | 78 | 30 | 4 | 37.0% | MH593359 |
| *F. yuzhongensis* | BM13-1 | 151,645 | 81,417 | 17,526 | 26,351 | 115 | 78 | 30 | 4 | 37.0% | MH244911 |
|  | BM13-2 | 151,645 | 81,417 | 17,526 | 26,351 | 115 | 78 | 30 | 4 | 37.0% | MN810969 |
|  | BM13-3 | 151,645 | 81,417 | 17,526 | 26,351 | 115 | 78 | 30 | 4 | 37.0% | MN810970 |
| *F. sinica* | BM14-1 | 152,064 | 81,827 | 17,537 | 26,350 | 115 | 78 | 30 | 4 | 36.9% | MH244912 |
|  | BM14-3 | 152,065 | 81,828 | 17,537 | 26,350 | 115 | 78 | 30 | 4 | 36.9% | MN810971 |
| *F. dajinensis* | BM15-1 | 151,991 | 81,723 | 17,540 | 26,364 | 115 | 78 | 30 | 4 | 36.9% | MH244913 |
|  | BM15-2 | 152,035 | 81,767 | 17,540 | 26,364 | 115 | 78 | 30 | 4 | 36.9% | MN810972 |
|  | BM15-3 | 151,991 | 81,723 | 17,540 | 26,364 | 115 | 78 | 30 | 4 | 36.9% | MN810973 |
| *F. thunbergii* | BM16-1 | 152,160 | 82,085 | 17,565 | 26,255 | 115 | 78 | 30 | 4 | 37.0% | MH244914 |
|  | BM16-2 | 152,160 | 81,895 | 17,565 | 26,350 | 115 | 78 | 30 | 4 | 37.0% | MH593360 |
|  | BM17-1 | 152,160 | 81,895 | 17,565 | 26,350 | 115 | 78 | 30 | 4 | 37.0% | MH593361 |
|  | BM17-2 | 152,160 | 81,895 | 17,565 | 26,350 | 115 | 78 | 30 | 4 | 37.0% | MH593362 |
| *F. monantha* | BM18-1 | 152,184 | 81,925 | 17,561 | 26,349 | 115 | 78 | 30 | 4 | 37.0% | MN810974 |
|  | BM18-2 | 152,182 | 81,923 | 17,559 | 26,350 | 115 | 78 | 30 | 4 | 37.0% | MN810975 |
|  | BM18-3 | 152,182 | 81,923 | 17,559 | 26,350 | 115 | 78 | 30 | 4 | 37.0% | MN810976 |
| *F. anhuiensis* | BM19-1 | 152,119 | 81,817 | 17,560 | 26,371 | 115 | 78 | 30 | 4 | 37.0% | MN810977 |
|  | BM19-2 | 152,119 | 81,817 | 17,560 | 26,371 | 115 | 78 | 30 | 4 | 37.0% | MN810978 |
|  | BM19-3 | 152,119 | 81,817 | 17,560 | 26,371 | 115 | 78 | 30 | 4 | 37.0% | MN810979 |
|  | BM20-1 | 152,119 | 81,817 | 17,560 | 26,371 | 115 | 78 | 30 | 4 | 37.0% | MN810980 |
|  | BM20-2 | 152,119 | 81,817 | 17,560 | 26,371 | 115 | 78 | 30 | 4 | 37.0% | MH593363 |
|  | BM20-3 | 152,119 | 81,817 | 17,560 | 26,371 | 115 | 78 | 30 | 4 | 37.0% | MN810981 |
| *F. davidii* | BM21-1 | 152,888 | 82,358 | 17,038 | 26,746 | 115 | 78 | 30 | 4 | 36.9% | MN810982 |
|  | BM21-2 | 152,888 | 82,453 | 17,038 | 26,651 | 115 | 78 | 30 | 4 | 36.9% | MN810983 |
|  | BM21-3 | 152,888 | 82,358 | 17,038 | 26,746 | 115 | 78 | 30 | 4 | 36.9% | MN810984 |
| *F. tortifolia* | BM22-1 | 152,018 | 81,788 | 17,510 | 26,360 | 115 | 78 | 30 | 4 | 37.0% | MN810985 |
|  | BM22-2 | 152,018 | 81,788 | 17,510 | 26,360 | 115 | 78 | 30 | 4 | 37.0% | MN810986 |
|  | BM22-3 | 151,943 | 81,714 | 17,511 | 26,359 | 115 | 78 | 30 | 4 | 37.0% | MN810987 |
| *F. pallidiflora* | BM23-1 | 152,073 | 81,779 | 17,514 | 26,390 | 115 | 78 | 30 | 4 | 37.0% | MH593364 |
|  | BM23-2 | 152,067 | 81,763 | 17,528 | 26,388 | 115 | 78 | 30 | 4 | 37.0% | MH593365 |
|  | BM23-3 | 152,073 | 81,780 | 17,513 | 26,390 | 115 | 78 | 30 | 4 | 37.0% | MH593366 |
| *F. walujewii* | BM24-1 | 151,923 | 81,706 | 17,521 | 26,348 | 115 | 78 | 30 | 4 | 36.9% | MN810988 |
|  | BM24-2 | 151,923 | 81,705 | 17,520 | 26,349 | 115 | 78 | 30 | 4 | 36.9% | MN810989 |
|  | BM25-1 | 151,921 | 81,698 | 17,521 | 26,351 | 115 | 78 | 30 | 4 | 36.9% | MN810990 |
|  | BM25-2 | 151,922 | 81,699 | 17,521 | 26,351 | 115 | 78 | 30 | 4 | 36.9% | MN810991 |
| *F. ussuriensis* | BM26-1 | 151,571 | 81,773 | 17,126 | 26,336 | 115 | 78 | 30 | 4 | 36.9% | MH593367 |
|  | BM26-2 | 151,523 | 81,741 | 17,122 | 26,330 | 115 | 78 | 30 | 4 | 37.0% | MH593368 |
|  | BM26-3 | 151,552 | 81,764 | 17,124 | 26,332 | 115 | 78 | 30 | 4 | 37.0% | MH593369 |
| *F. maximowiczii* | BM27-1 | 151,715 | 80,990 | 17,565 | 26,580 | 115 | 78 | 30 | 4 | 37.1% | MN810992 |
|  | BM27-2 | 151,711 | 80,988 | 17,563 | 26,580 | 115 | 78 | 30 | 4 | 37.1% | MN810993 |
|  | BM27-3 | 151,715 | 80,990 | 17,565 | 26,580 | 115 | 78 | 30 | 4 | 37.1% | MN810994 |
| *F. omeiensis* | BM28-1 | 152,019 | 81,768 | 17,551 | 26,350 | 115 | 78 | 30 | 4 | 36.9% | MN810995 |
|  | BM28-2 | 152,019 | 81,768 | 17,551 | 26,350 | 115 | 78 | 30 | 4 | 36.9% | MN810996 |
|  | BM28-3 | 152,019 | 81,768 | 17,551 | 26,350 | 115 | 78 | 30 | 4 | 36.9% | MN810997 |
| *F. crassicaulis* | BM29-1 | 151,860 | 81,615 | 17,547 | 26,349 | 115 | 78 | 30 | 4 | 37.0% | MN810998 |
|  | BM29-2 | 151,860 | 81,615 | 17,547 | 26,349 | 115 | 78 | 30 | 4 | 37.0% | MN810999 |
|  | BM29-3 | 151,860 | 81,615 | 17,547 | 26,349 | 115 | 78 | 30 | 4 | 37.0% | MN811100 |
| *F. hupehensis* | BM30-1 | 152,189 | 81,928 | 17,557 | 26,352 | 115 | 78 | 30 | 4 | 37.0% | MN811101 |
|  | BM30-2 | 152,189 | 81,928 | 17,557 | 26,352 | 115 | 78 | 30 | 4 | 37.0% | MN811102 |
|  | BM30-3 | 152,189 | 81,928 | 17,557 | 26,352 | 115 | 78 | 30 | 4 | 37.0% | MN811103 |
