# Supplementary material for: DNA Barcoding and Phylogenomic Analysis of the Genus Fritillaria in China Based on Complete Chloroplast Genomes
Source: Front Plant Sci. 2022 Feb 25;13:764255. doi: 10.3389/fpls.2022.764255 (PMC8914171; doi:10.3389/fpls.2022.764255)
Supplement: Supplementary Figure 1 — Plant morphology of the Fritillaria species in this study. [file Data_Sheet_1.zip › Table S4.DOCX]

**SUPPLEMENTARY TABLE 4 |** Gene contents of chloroplast genome in *Fritillaria*.

| Category for gene | Group of genes | Name of genes |
| --- | --- | --- |
| Self-replication | Large subunit of ribosome | *rpl2*^a^*, *rpl14*, *rpl16**, *rpl20*, *rpl22*, *rpl23*^a^, *rpl32*, *rpl33*, *rpl36* |
|  | Small subunit of ribosome | *rps2*, *rps3*, *rps4*, *rps7*^a^, *rps8*, *rps11*, *rps12*^a^*, *rps14*, *rps15*, *rps16**, *rps18*, *rps19* |
|  | DNA dependent RNA polymerase | *rpoA*, *rpoB*, *rpoC1**, *rpoC2* |
|  | rRNA gene | *rrn4.5*^a^, *rrn5*^a^, *rrn16*^a^, *rrn23*^a^ |
|  | tRNA gene | *trnK-UUU**, *trnI-GAU*^a^*, *trnA-UGC*^a^*, *trnG-GCC**, *trnV-UAC**, *trnL-UAA**, *trnS-UGA*, *trnS-GCU*, *trnS-GGA*, *trnY-GUA*, *trnC-GCA*, *trnL-CAA*^a^, *trnL-UAG*, *trnH-GUG*^a^, *trnD-GUC*, *trnfM-CAU*, *trnW-CCA*, *trnP-UGG*, *trnI-CAU*^a^, *trnR-ACG*^a^, *trnI-CAU*^a^, *trnE-UUC*, *trnT-UGU*, *trnF-GAA*, *trnQ-UUG*, *trnR-UCU*, *trnT-GGU*, *trnM-CAU*, *trnV-GAC*^a^, *trnN-GUU*^a^, *trnN-GUU*^a^, *trnV-GAC*^a^, *trnG-UCC* |
| Gene for photosynthesis | Subunits of photosystemⅠ | *psaA*, *psaB*, *psaC*, *psaI*, *psaJ* |
|  | Subunits of photosystem Ⅱ | *psbA*, *psbB*, *psbC*, *psbD*, *psbE*, *psbF*, *psbH*, *psbI*, *psbJ*, *psbK*, *psbL*, *psbM*, *psbN*, *psbT*, *psbZ* |
|  | Subunits of NADH-dehydrogenase | *ndhA**, *ndhB*^a^*, *ndhC*, *ndhD*, *ndhE*, *ndhF*, *ndhG*, *ndhH*, *ndhI*, *ndhJ*, *ndhK* |
|  | Subunits of cytochrome b/f complex | *petA*, *petB**, *petD**, *petG*, *petL*, *petN* |
|  | Subunit for ATP synthase | *atpA*, *atpB*, *atpE*, *atpF**, *atpH*, *atpI* |
|  | Large subunit of rubisco | *rbcL* |
| Other genes | Translational initiation factor | *infA* |
|  | Maturase | *matK* |
|  | Protease | *clpP** |
|  | Envelope membrane protein | *cemA* |
|  | Subunit of Acetyl-carboxylase | *accD* |
|  | C-type cytochrome synthesis gene | *ccsA* |
|  | Open reading frames (ORF,ycf) | *ycf1*, *ycf2*^a^, *ycf3**, *ycf4*, *ycf15*^a^, *ycf68*^a^ |

The “a” label after gene names reflect genes located in IR regions. Intron containing gene is indicated by one asterisk.
