# Supplementary figures and images for: DNA Barcoding and Phylogenomic Analysis of the Genus Fritillaria in China Based on Complete Chloroplast Genomes
Source: Front Plant Sci. 2022 Feb 25;13:764255. doi: 10.3389/fpls.2022.764255 (PMC8914171; doi:10.3389/fpls.2022.764255)

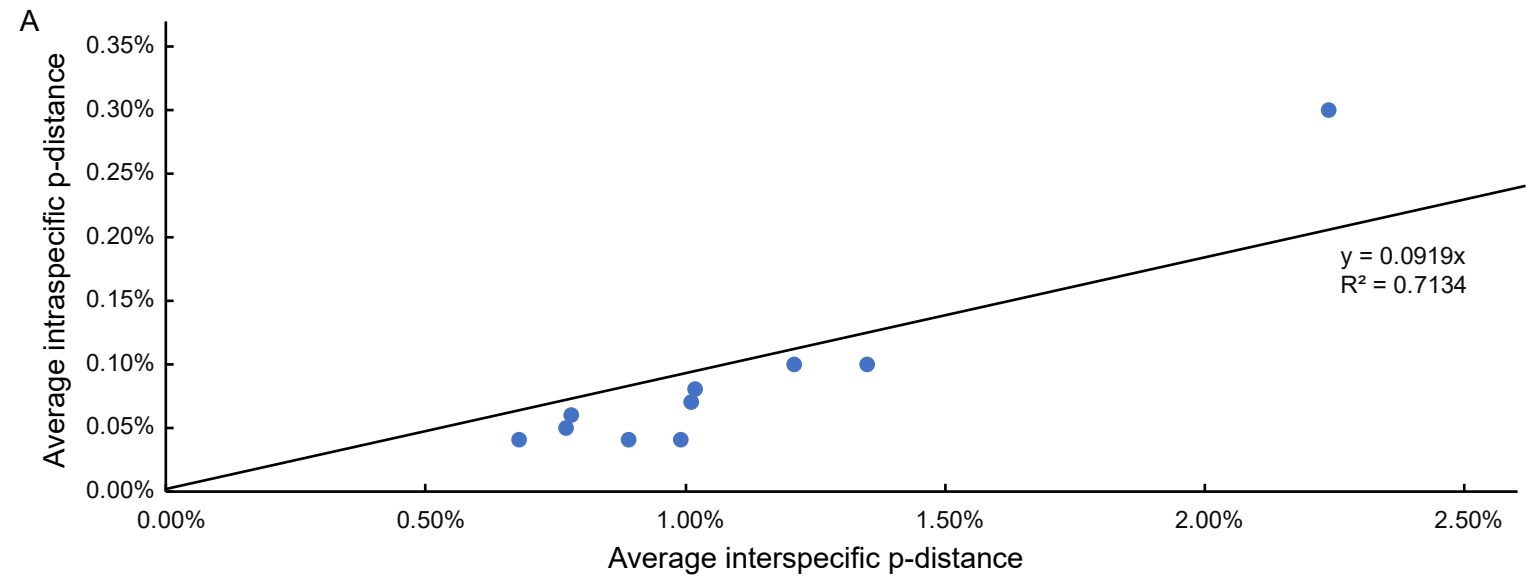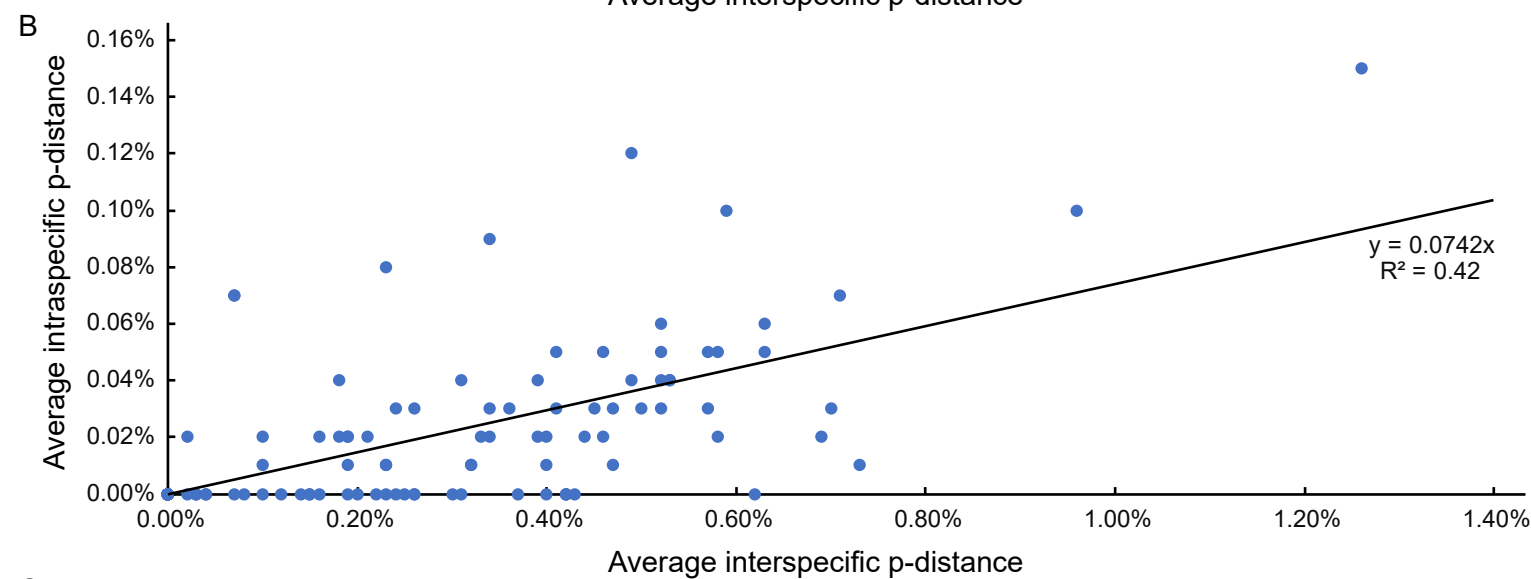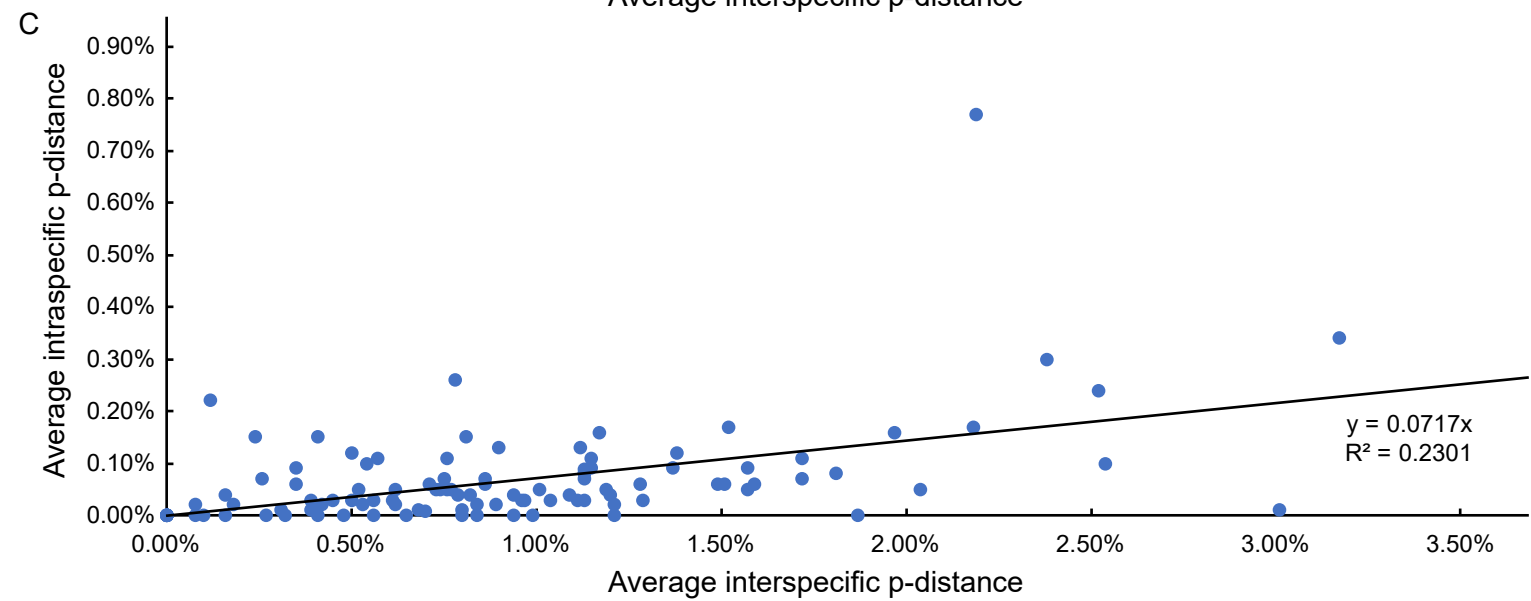

Supplement: Supplementary Figure 1 — Plant morphology of the Fritillaria species in this study. [file Data_Sheet_1.zip › Figure S3.PDF]

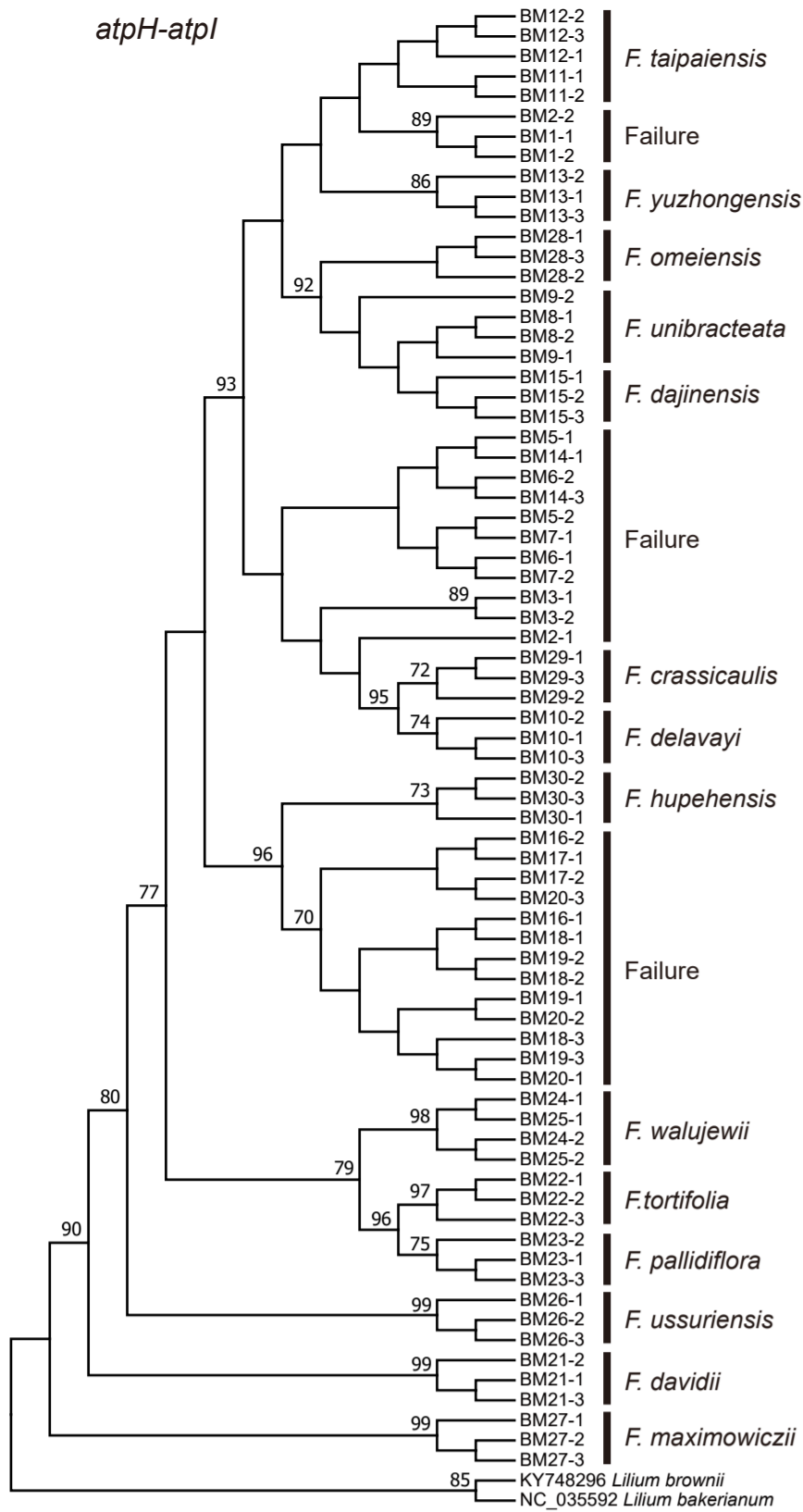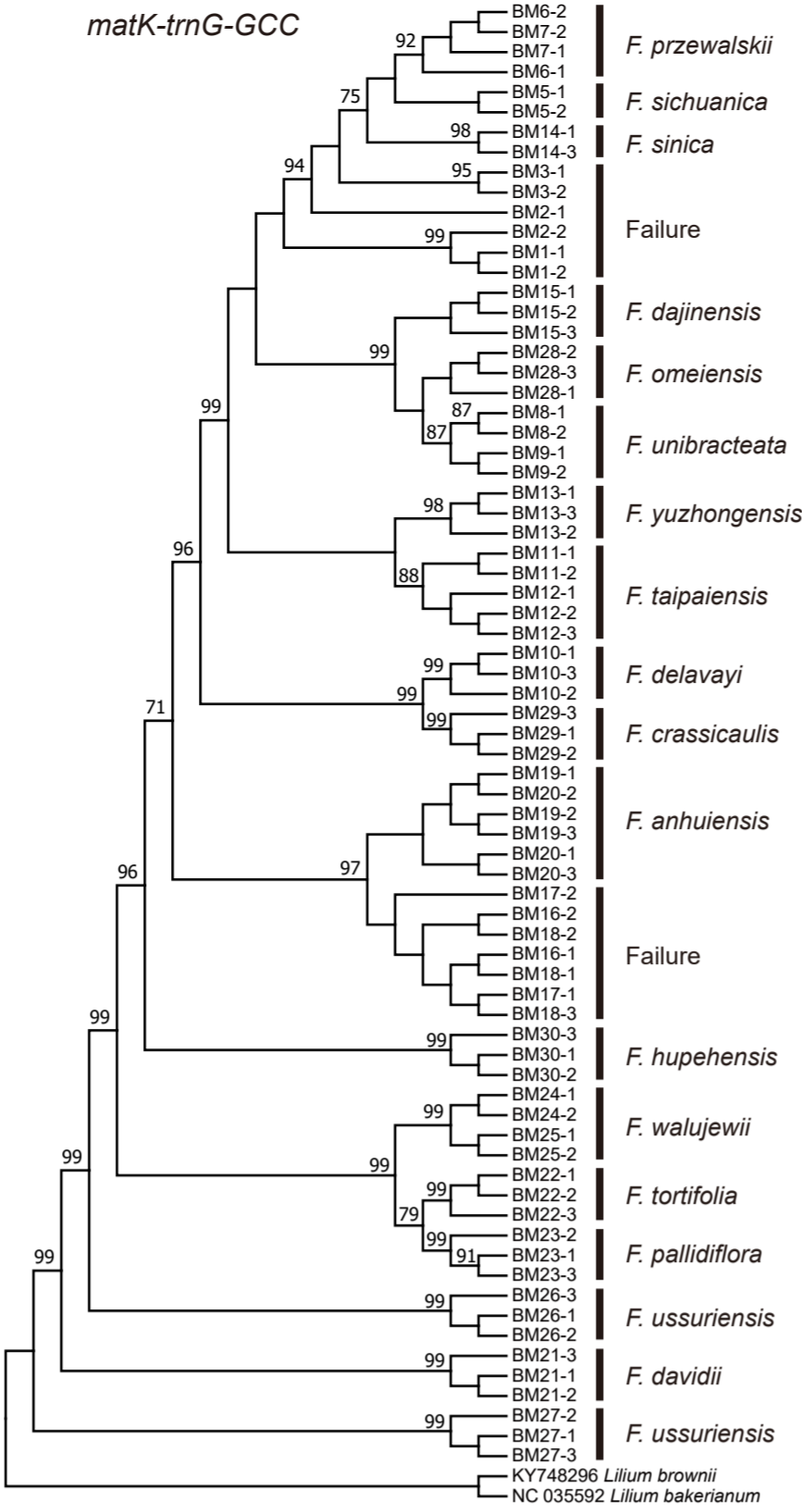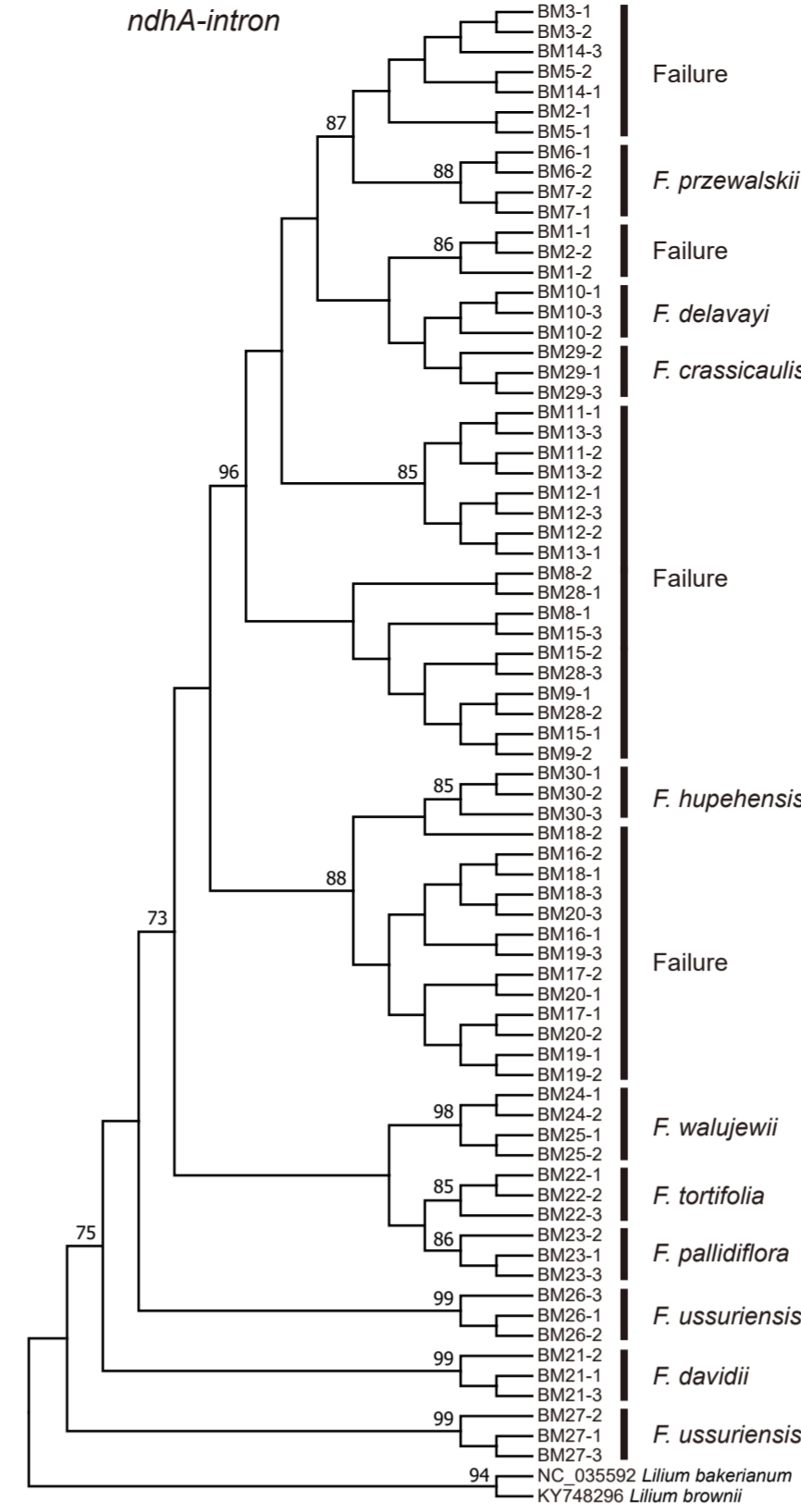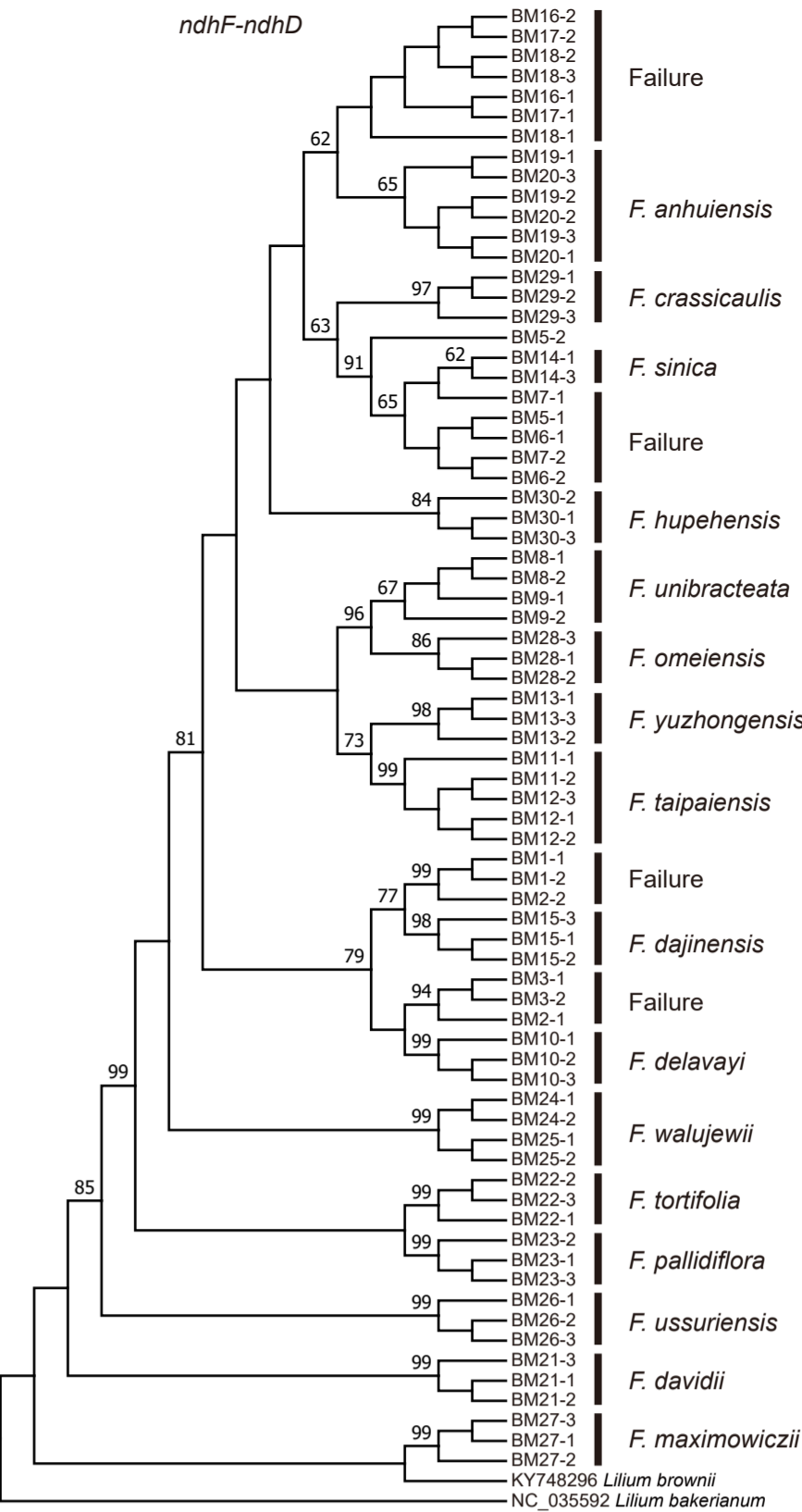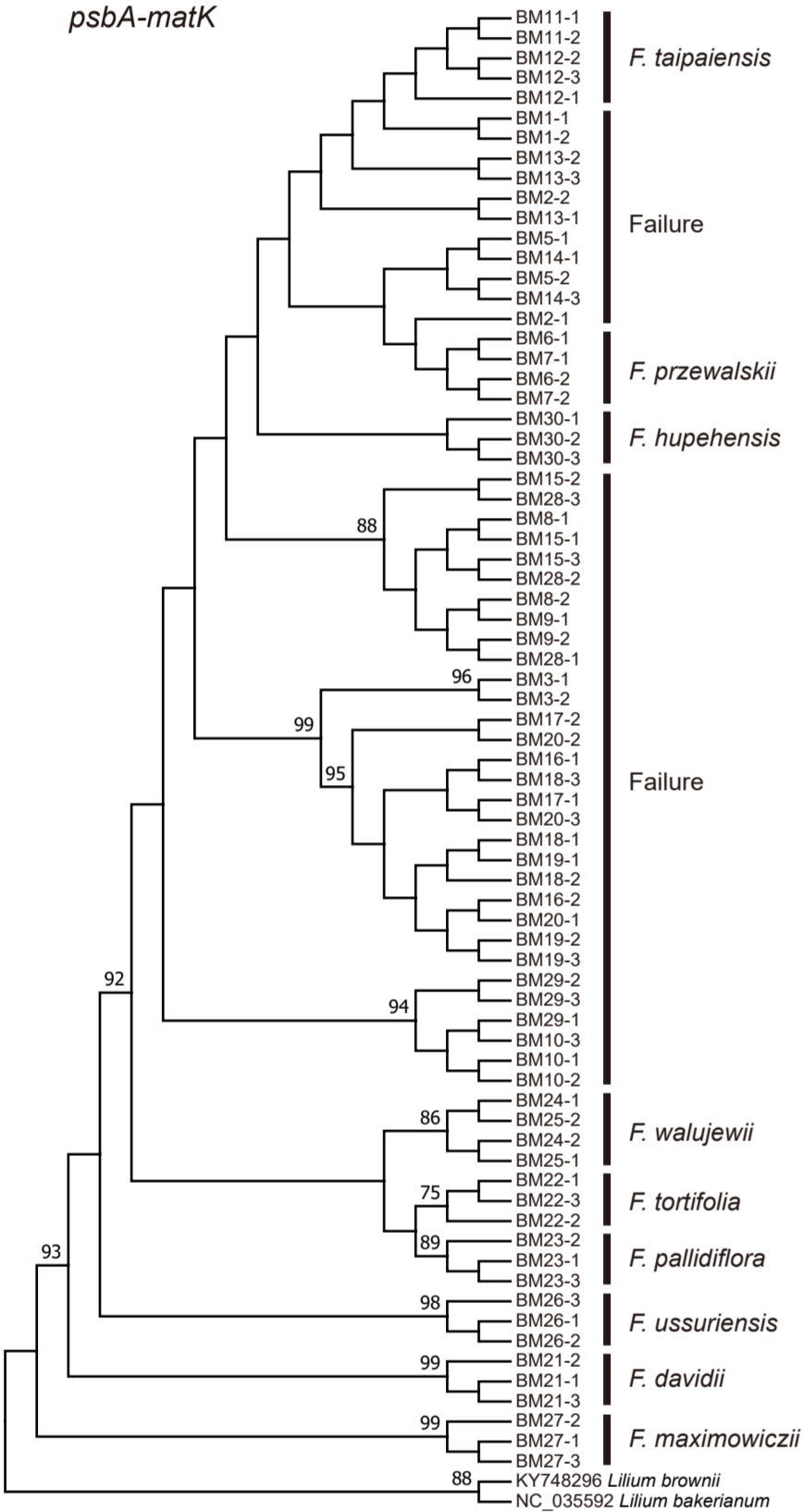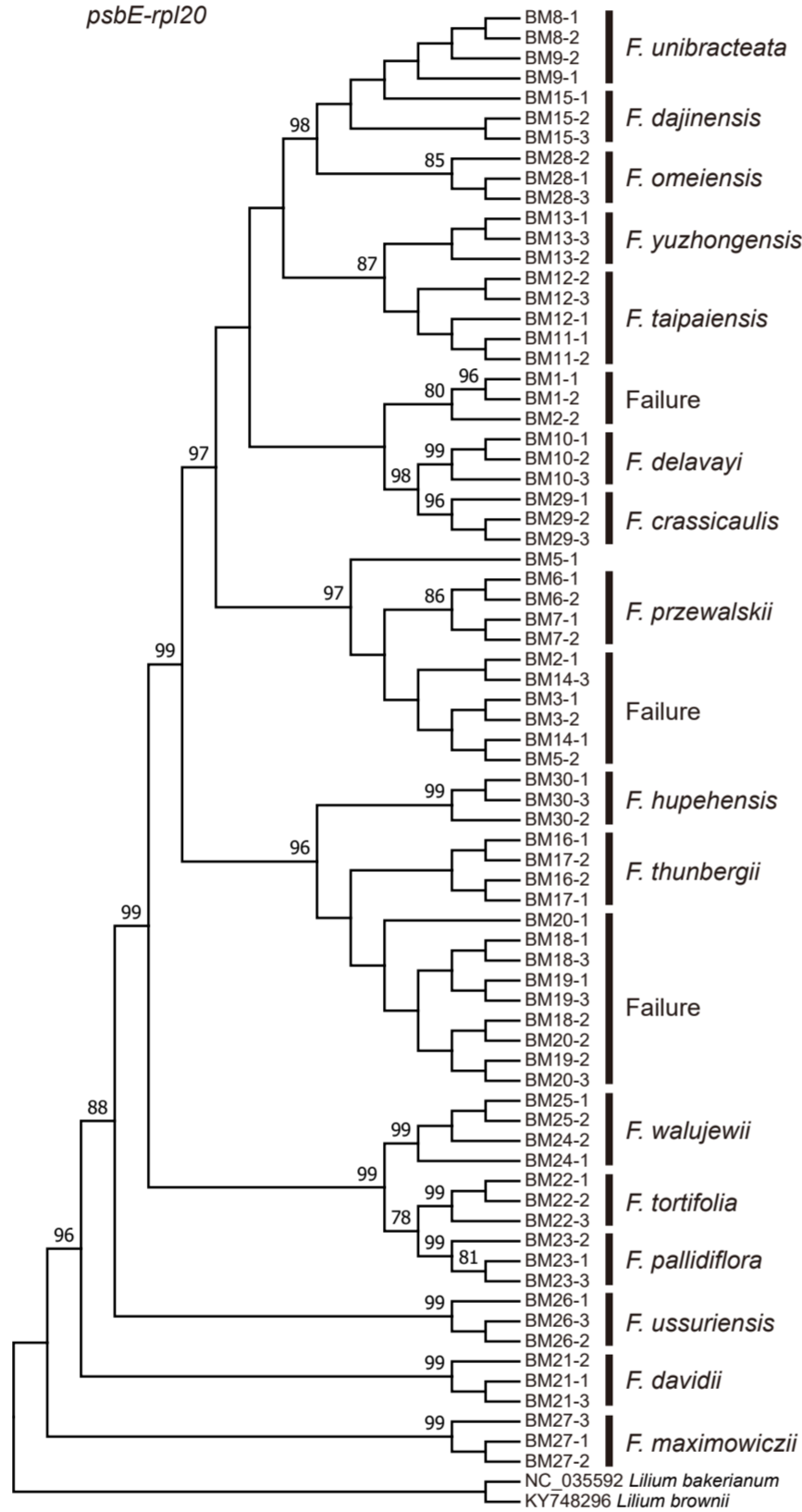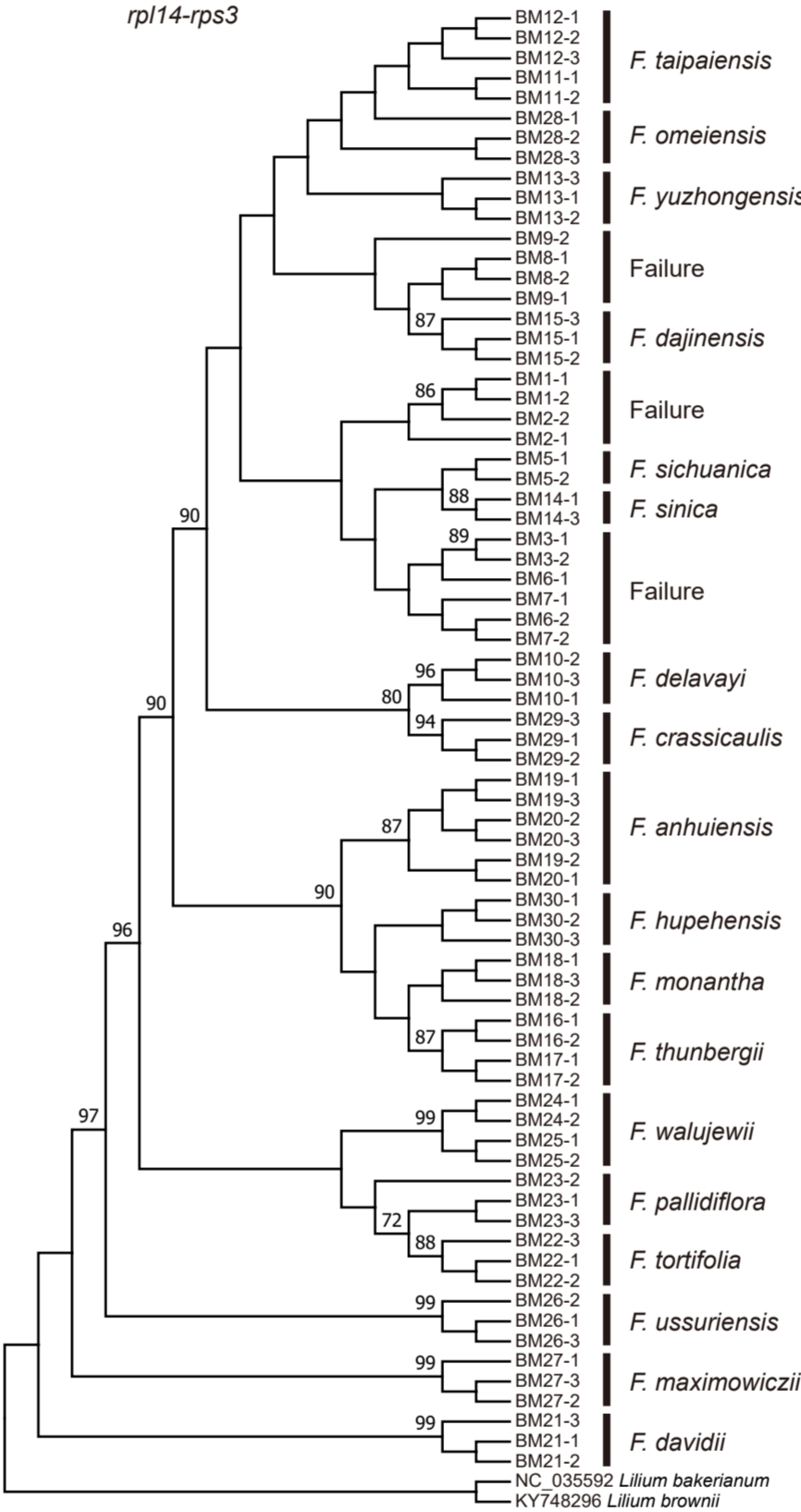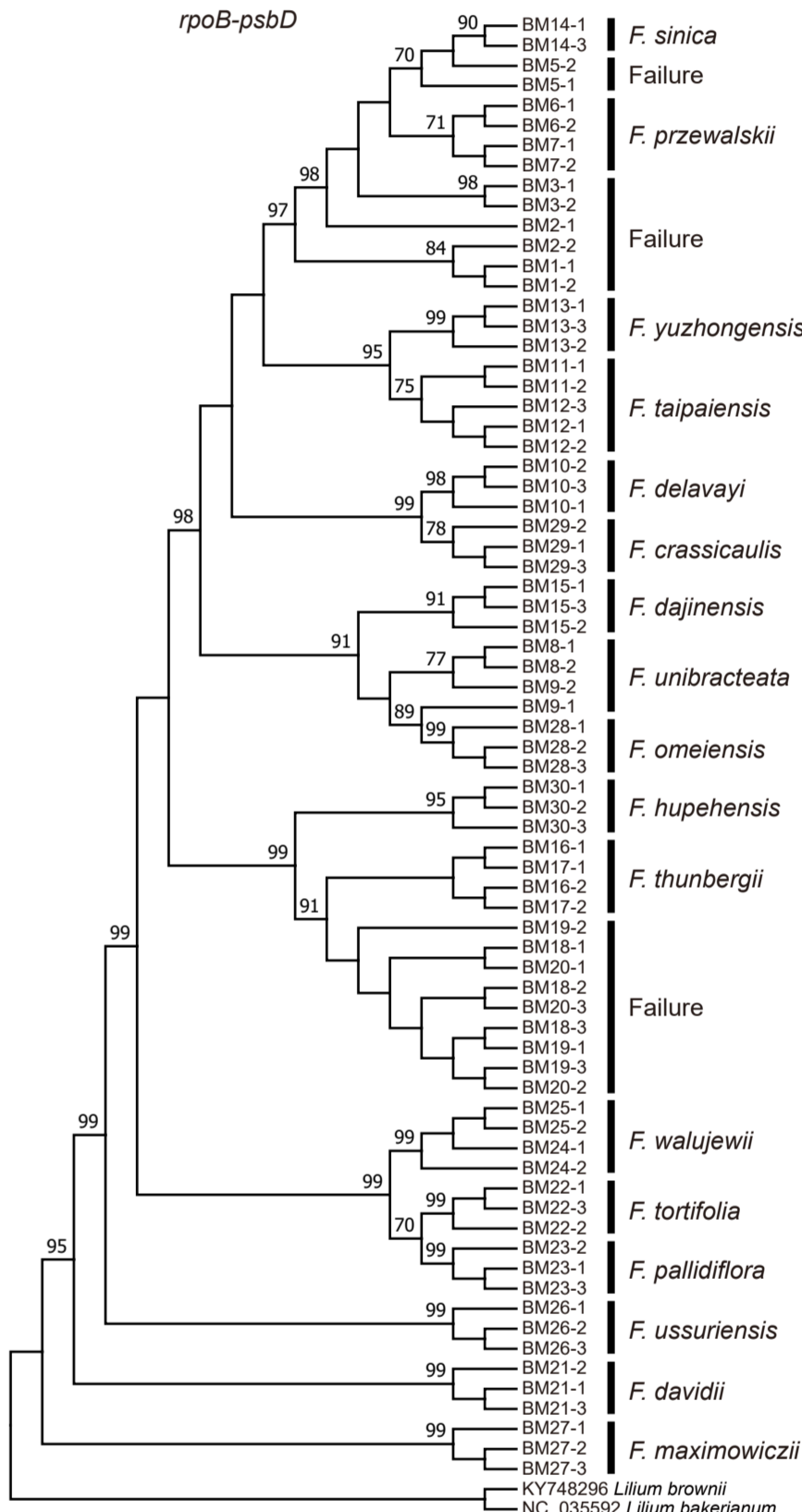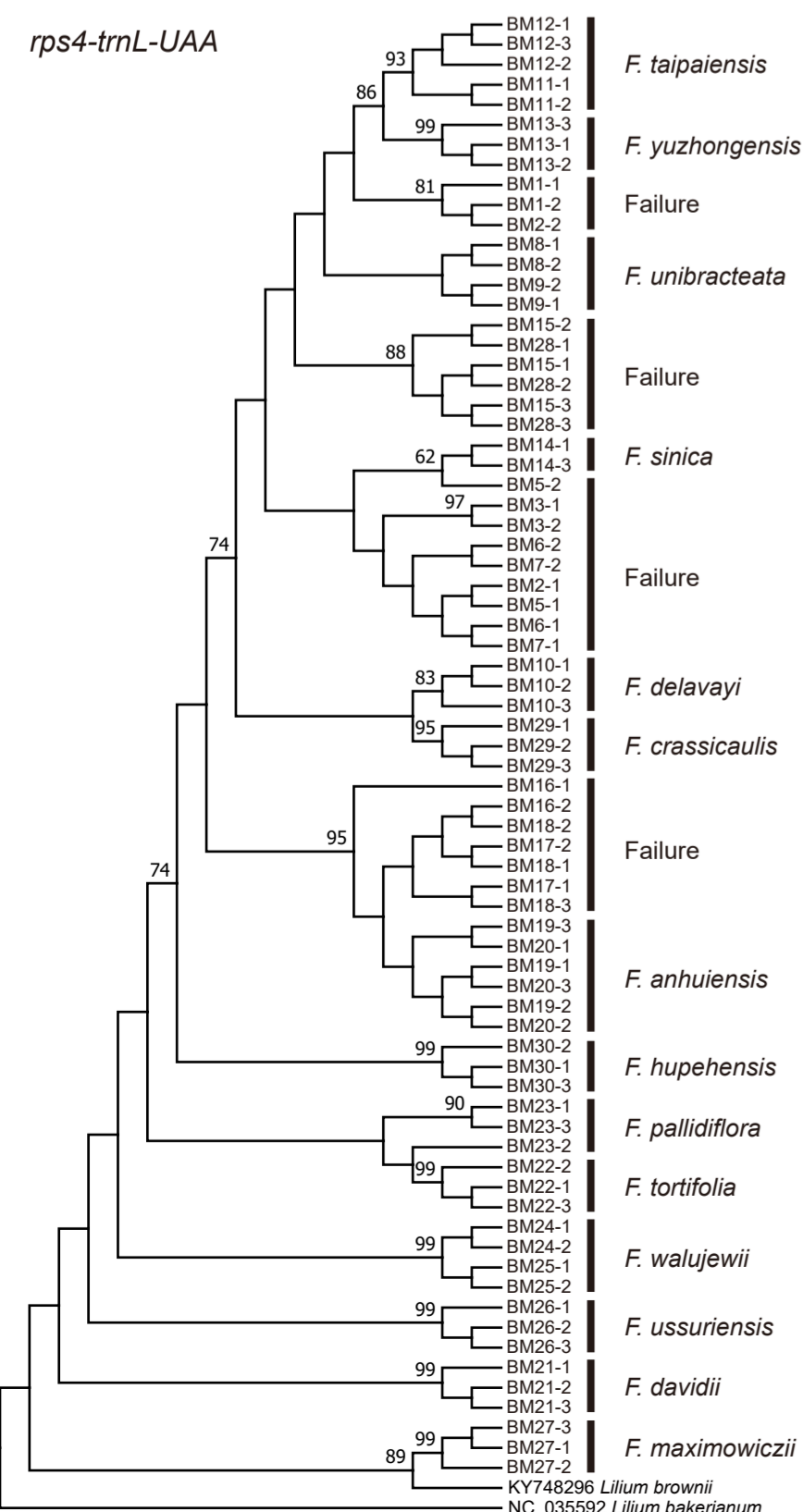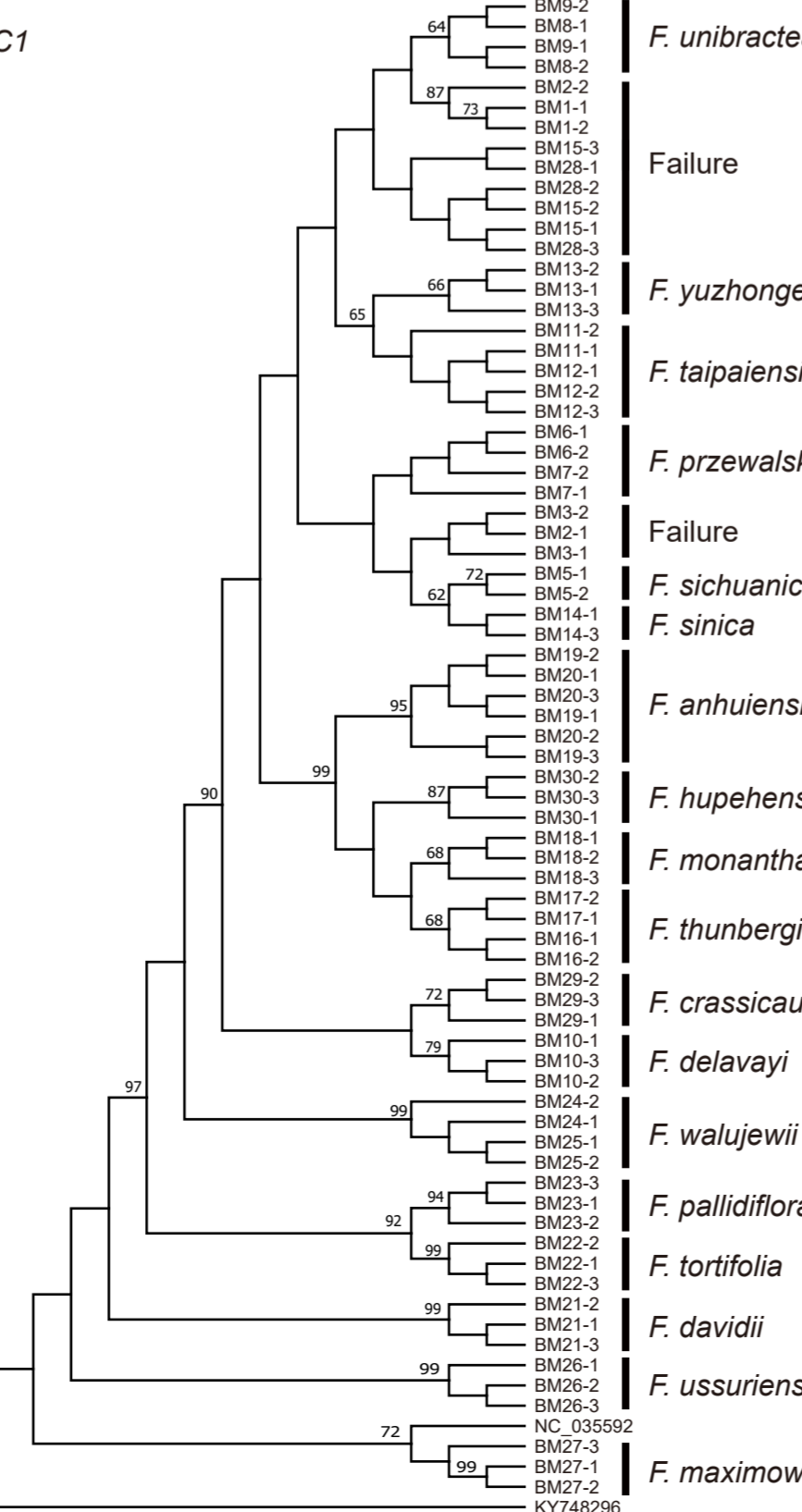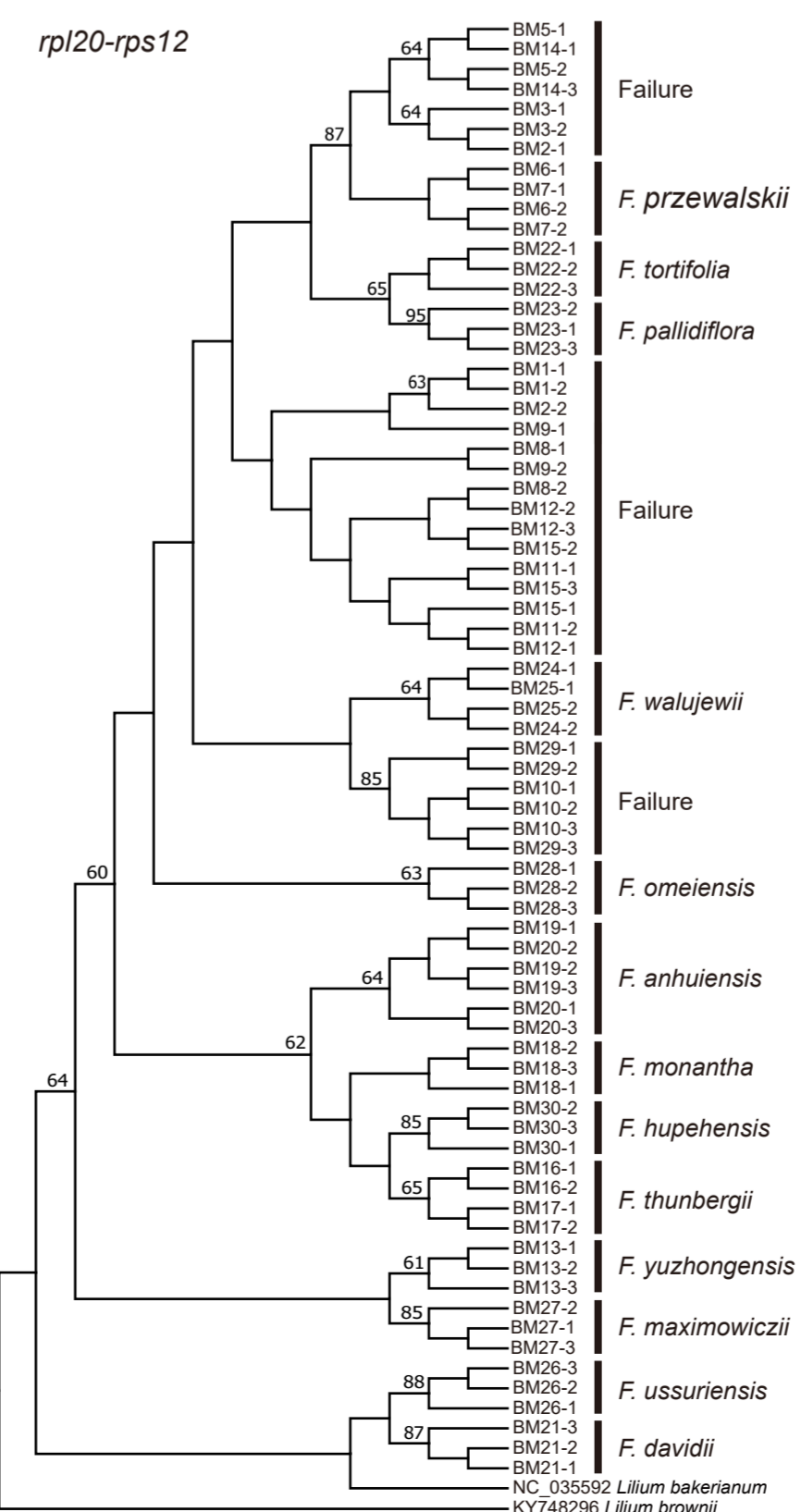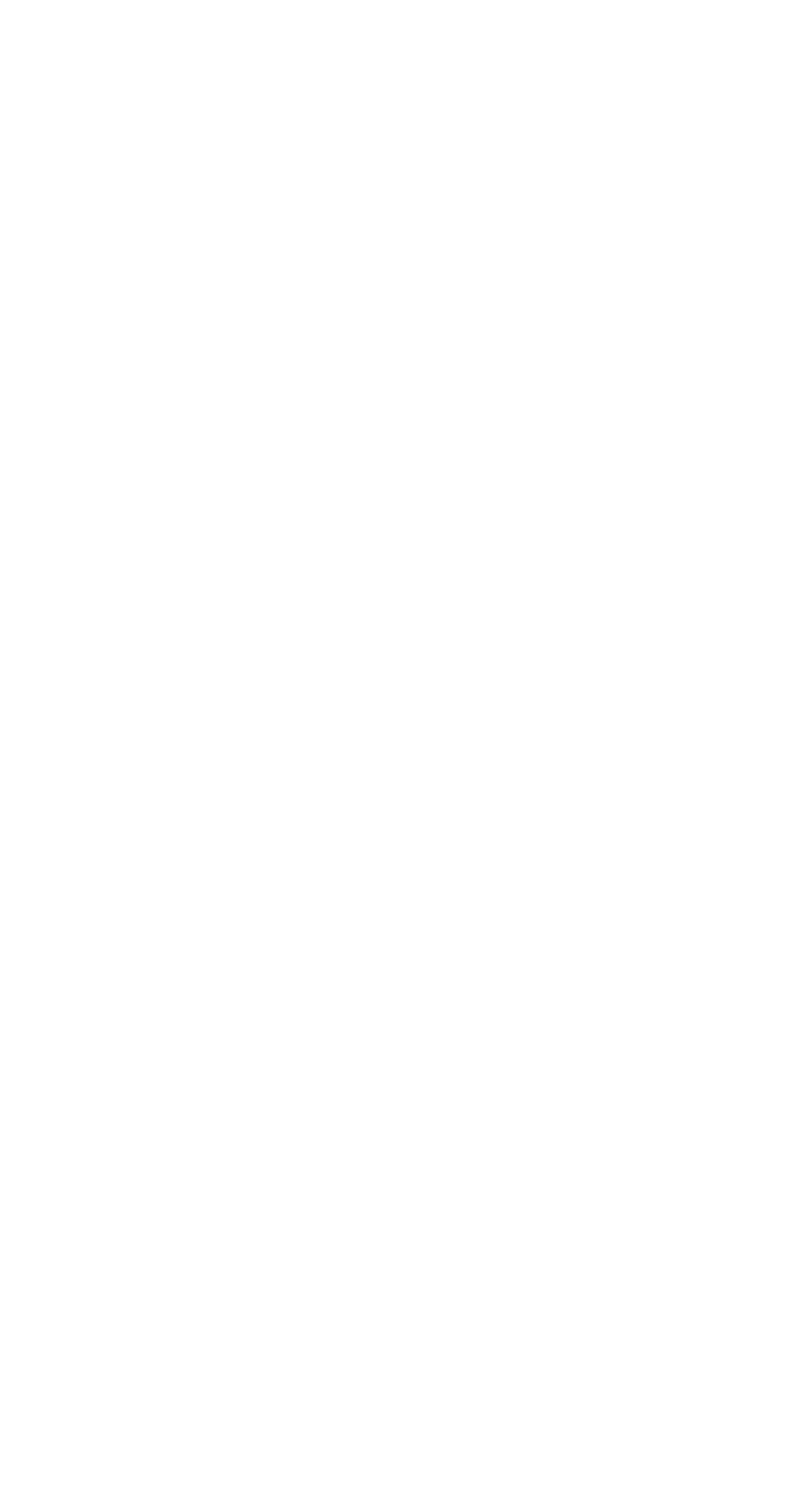

Supplement: Supplementary Figure 1 — Plant morphology of the Fritillaria species in this study. [file Data_Sheet_1.zip › Figure S4.PDF]

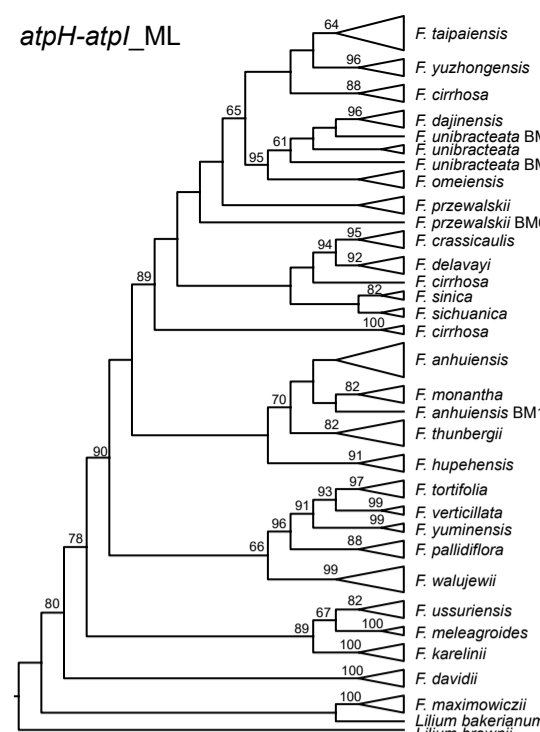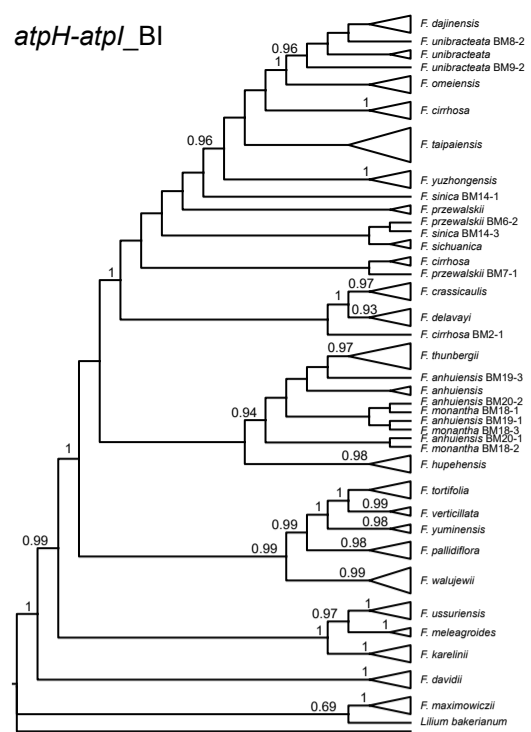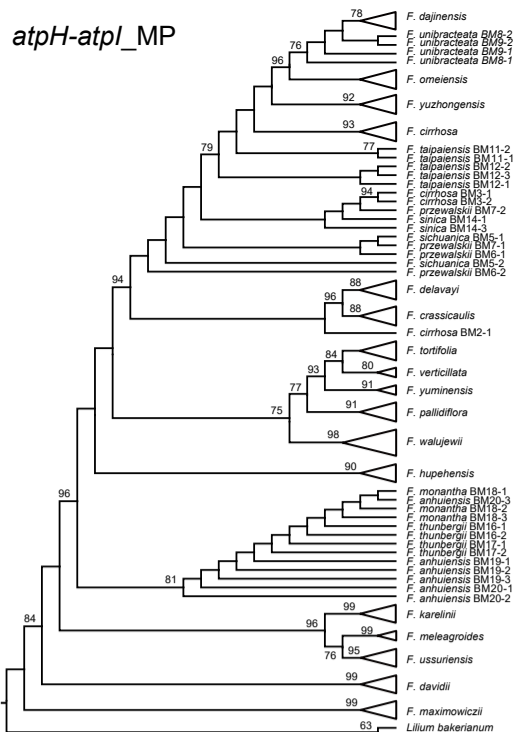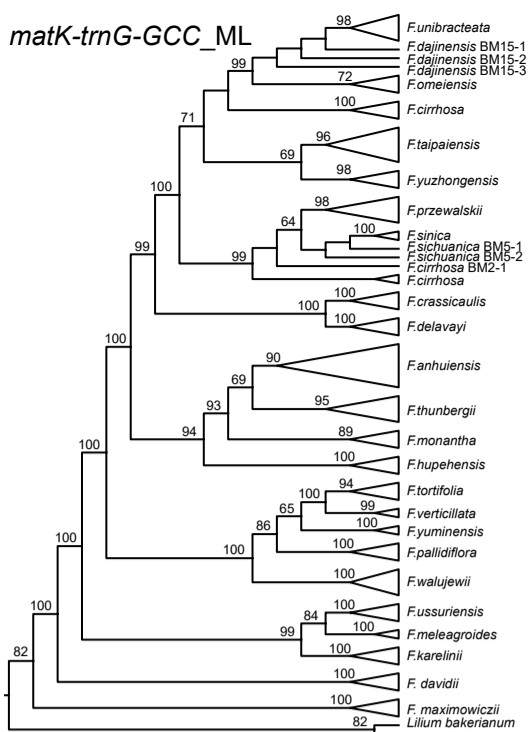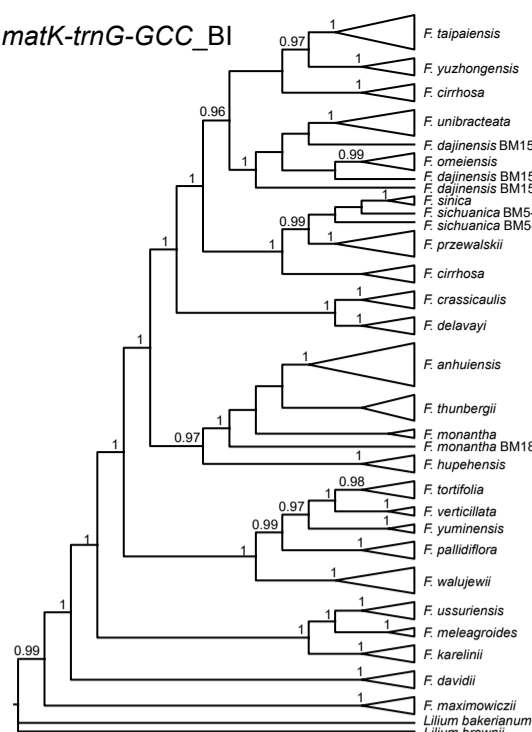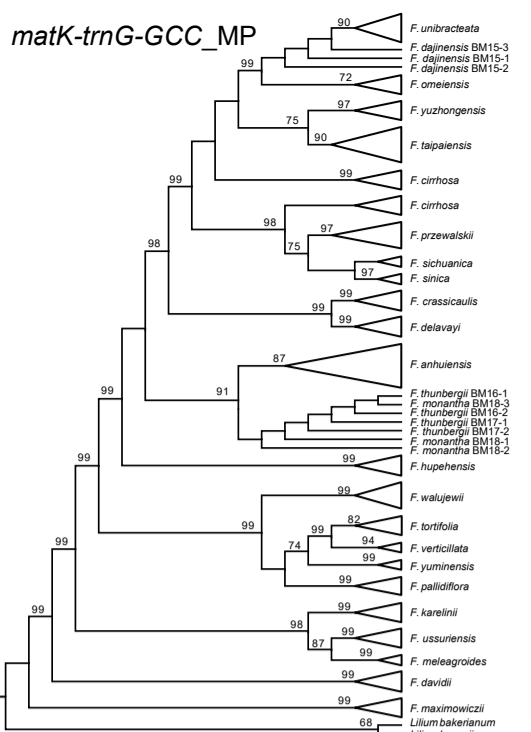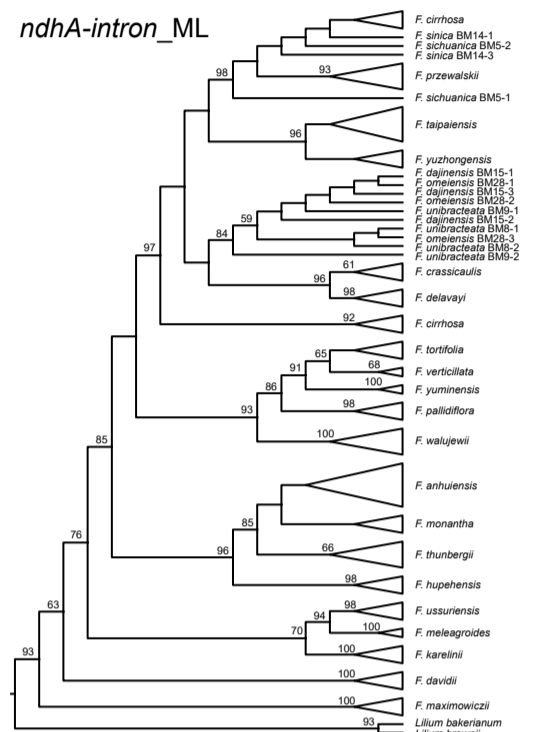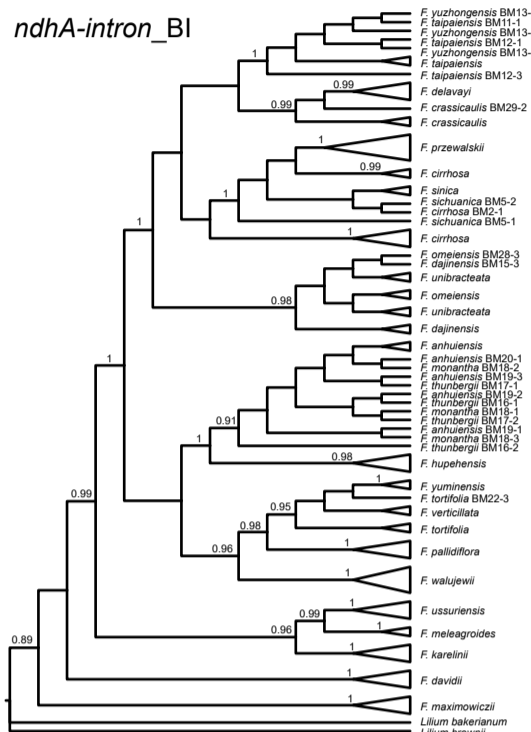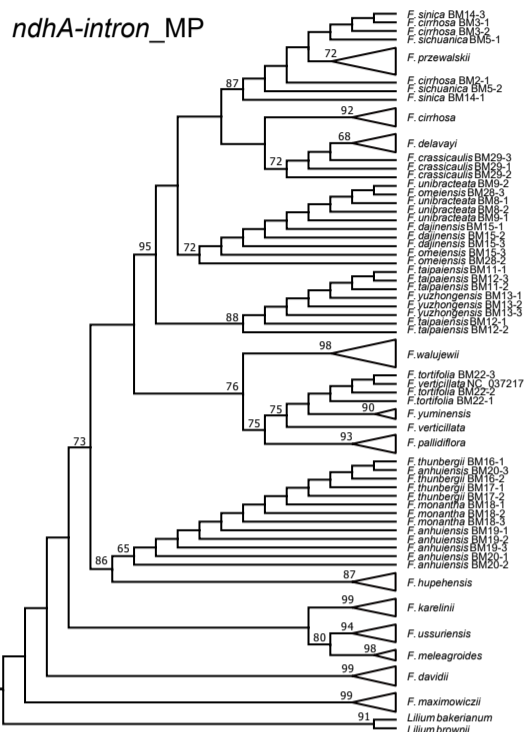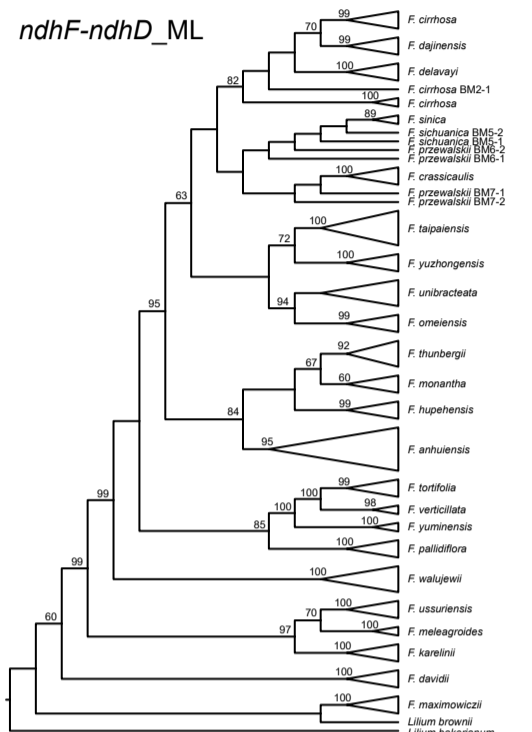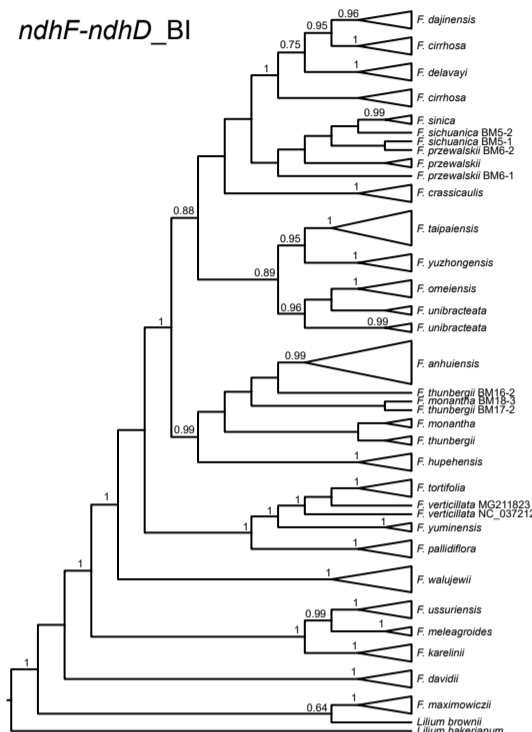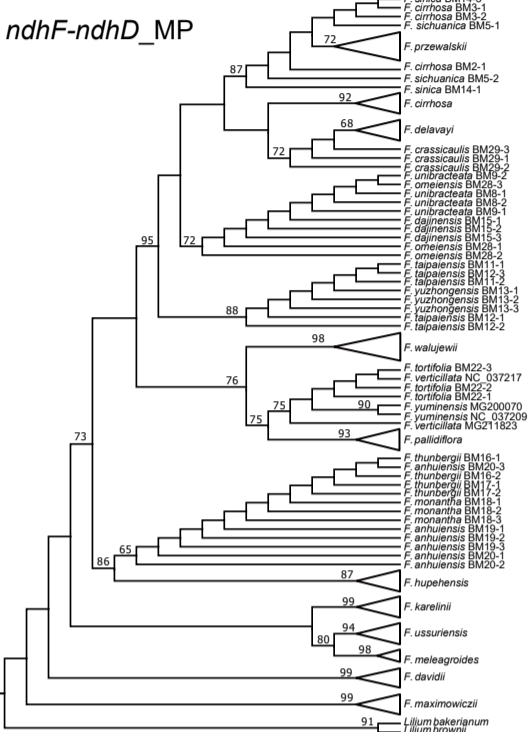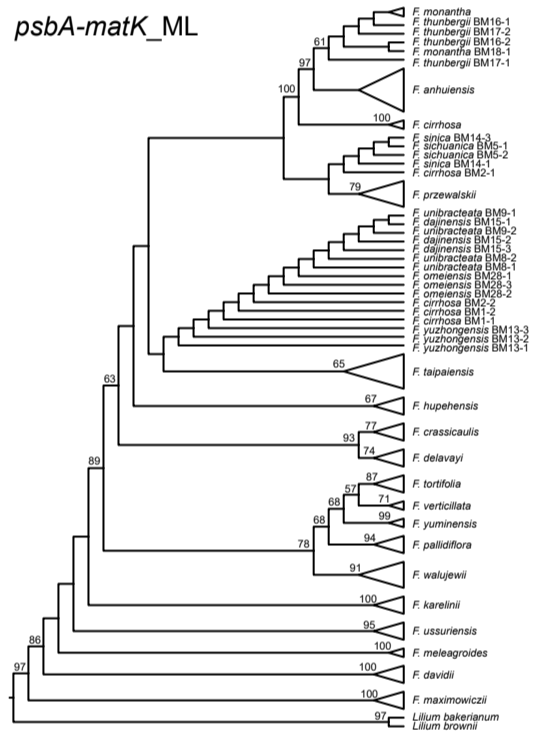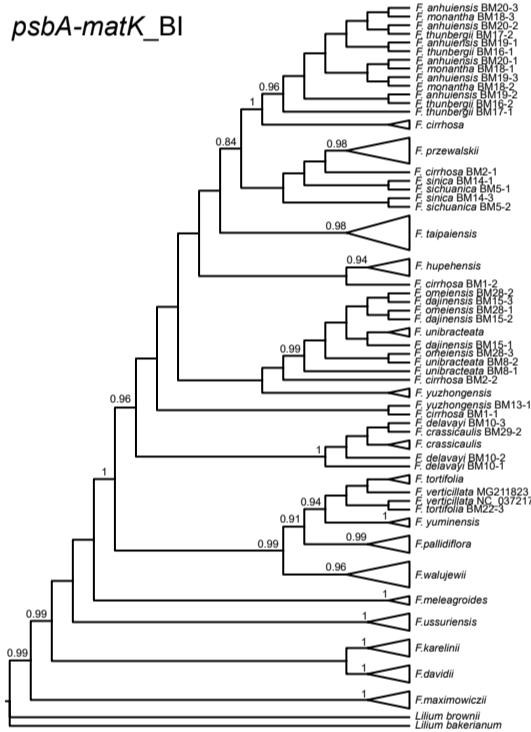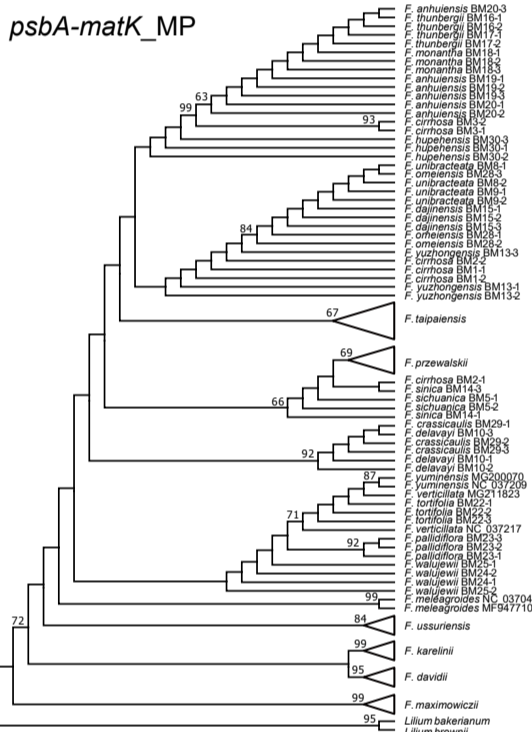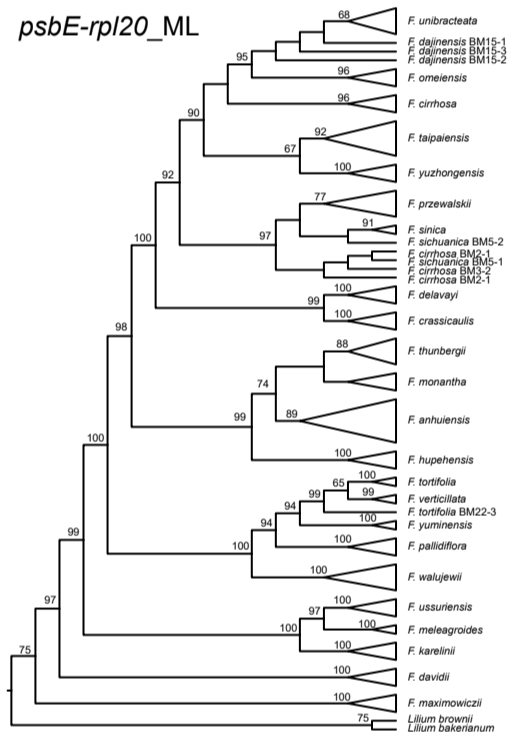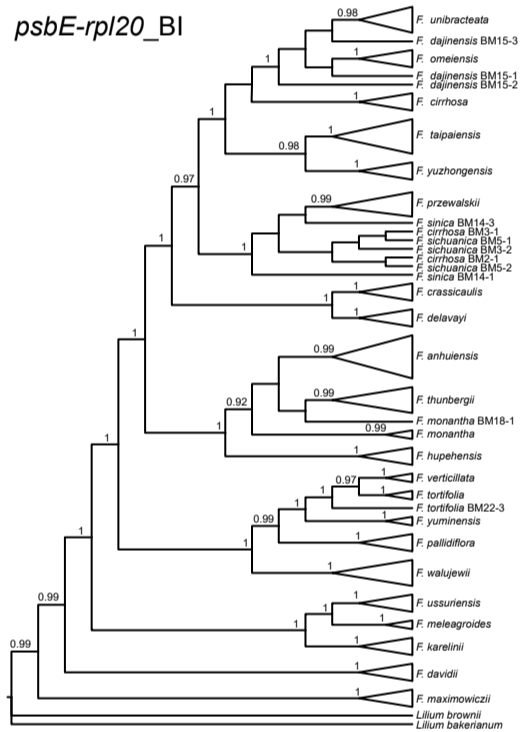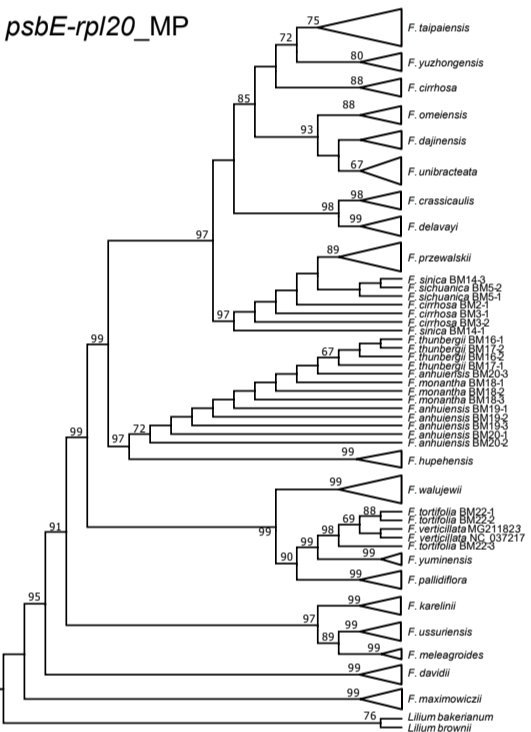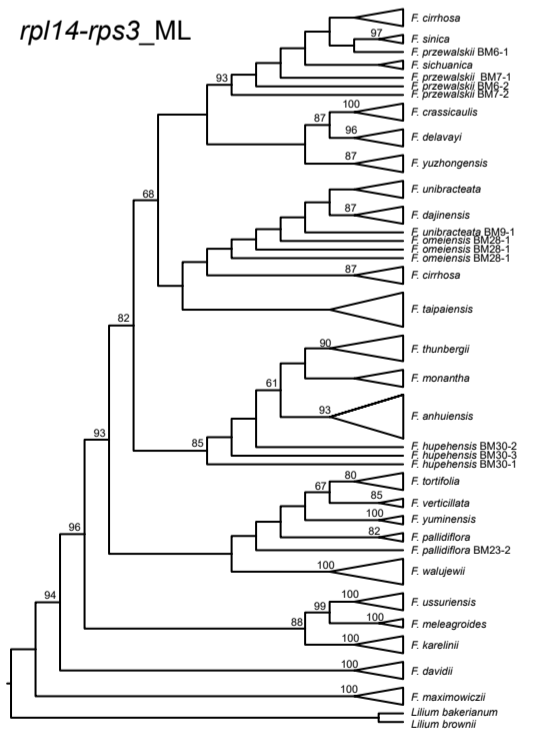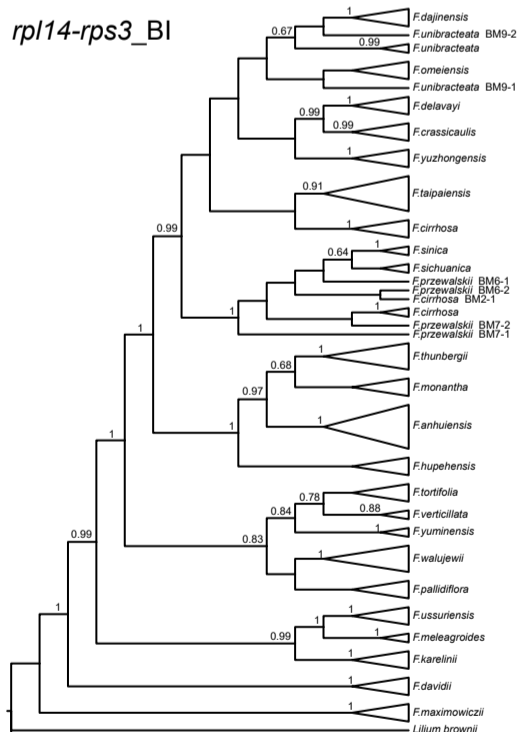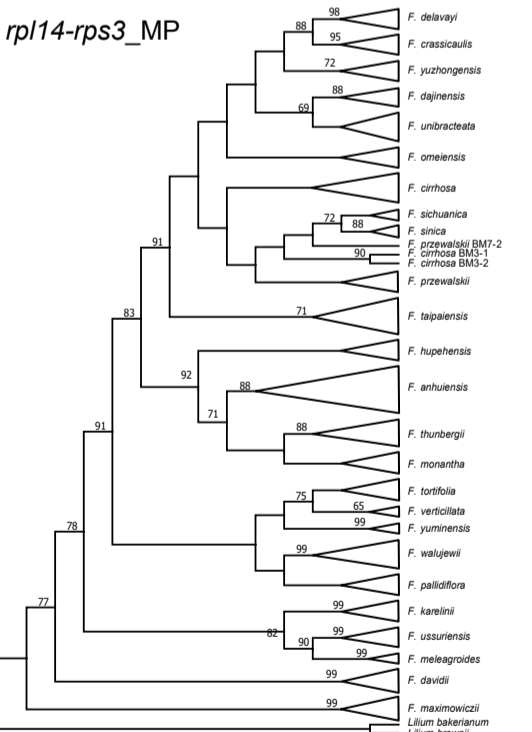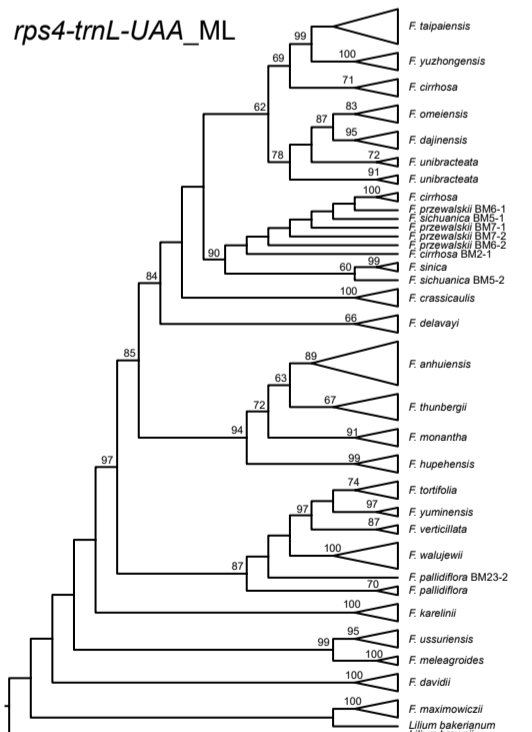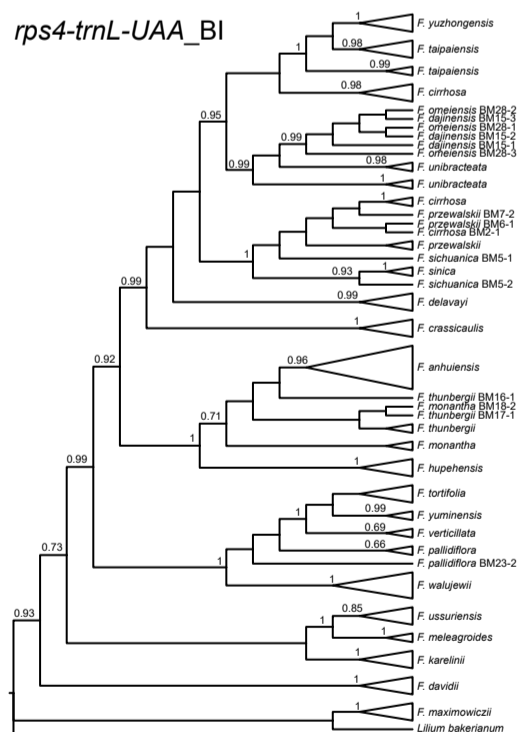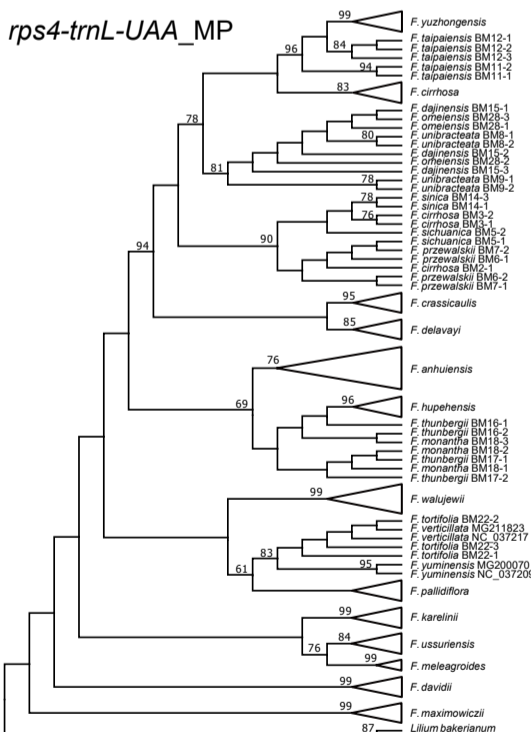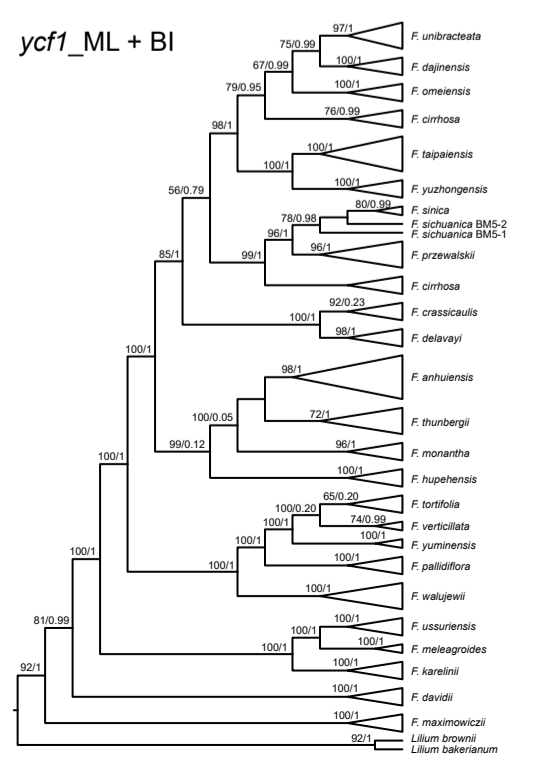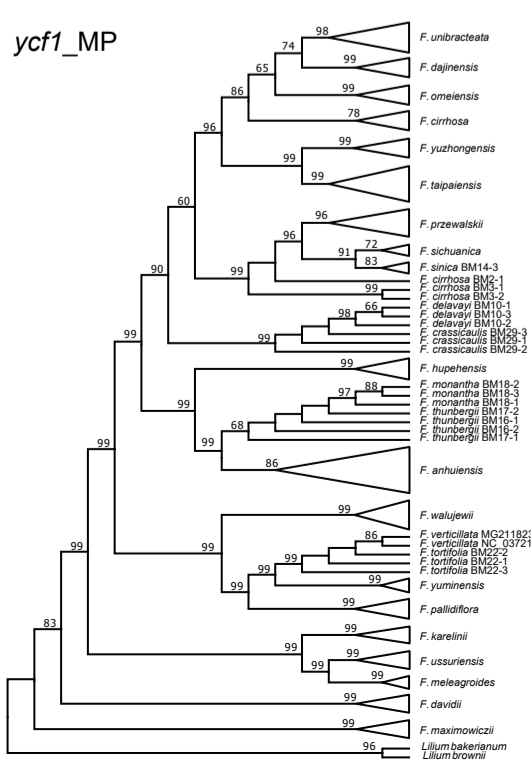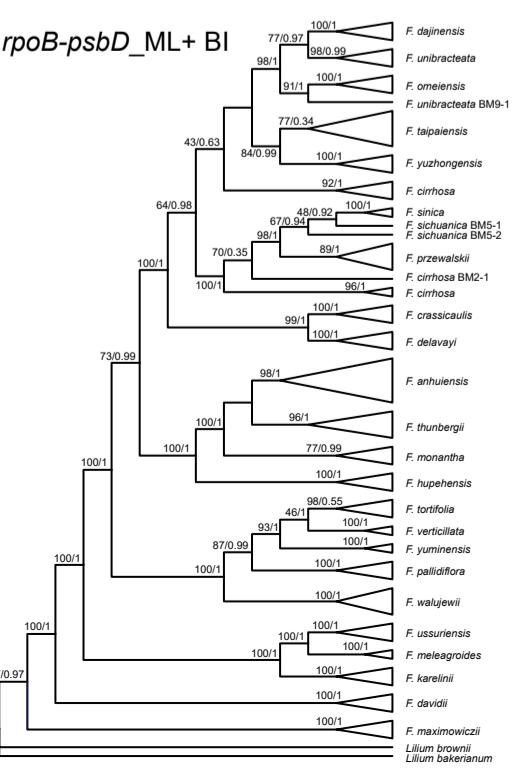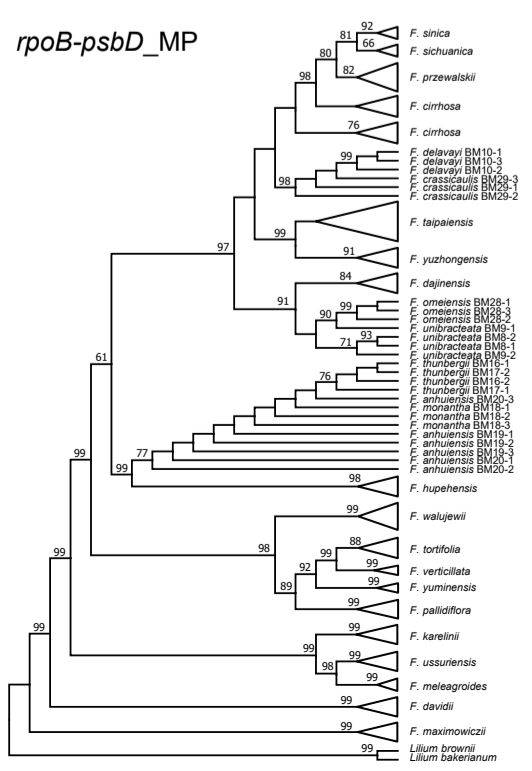

Supplement: Supplementary Figure 1 — Plant morphology of the Fritillaria species in this study. [file Data_Sheet_1.zip › Figure S5.PDF]
